# PreImplantation factor (PIF) therapy provides comprehensive protection against radiation induced pathologies

**DOI:** 10.18632/oncotarget.10635

**Published:** 2016-07-16

**Authors:** Reut Shainer, Osnat Almogi-Hazan, Arye Berger, Liad Hinden, Martin Mueller, Chaya Brodie, Cedric Simillion, Michael Paidas, Eytan R. Barnea, Reuven Or

**Affiliations:** ^1^ Department of Bone Marrow Transplantation and Cancer Immunotherapy, Hadassah-Hebrew University Medical Center, Jerusalem, 91120, Israel; ^2^ Department of Obstetrics, Gynecology, and Reproductive Sciences, Yale University School of Medicine, New Haven, CT 06510, USA; ^3^ Department of Obstetrics and Gynecology, University Hospital Bern, Bern, 3003, Switzerland; ^4^ Henry Ford Hospital, Detroit, MI 48202, USA; ^5^ Department of Clinical Research, University of Bern, Bern, 3003, Switzerland; ^6^ The Society for The Investigation of Early Pregnancy (SIEP), Cherry Hill, NJ 08003, USA; ^7^ BioIncept, LLC (PreImplantation Factor* Proprietary), Cherry Hill, NJ 08003, USA

**Keywords:** ARS, PreImplantation Factor (PIF), oxidative stress, local & systemic protection

## Abstract

Acute Radiation Syndrome (ARS) may lead to cancer and death and has few effective countermeasures. Efficacy of synthetic PIF treatment was demonstrated in preclinical autoimmune and transplantation models. PIF protected against inflammation and mortality following lethal irradiation in allogeneic bone marrow transplant (BMT) model. Herein, we demonstrate that PIF imparts comprehensive local and systemic protection against lethal and sub-lethal ARS in murine models. PIF treatment 2 h after lethal irradiation led to 100% survival and global hematopoietic recovery at 2 weeks after therapy. At 24 h after irradiation PIF restored hematopoiesis in a semi-allogeneic BMT model. PIF-preconditioning provided improved long-term engraftment. The direct effect of PIF on bone marrow cells was also demonstrated *in vitro*: PIF promoted pre-B cell differentiation and increased immunoregulatory properties of BM-derived mesenchymal stromal cells. PIF treatment also improved hematopoietic recovery and reduced systemic inflammatory cytokine production after sub-lethal radiation exposure. Here, PIF also prevented colonic crypt and basal membrane damage coupled with reduced nitric oxide synthetase (*iNOS*) and increased (B7h1) expression. Global upper GI gene pathway analysis revealed PIF's involvement in protein-RNA interactions, mitochondrial oxidative pathways, and responses to cellular stress. Some effects may be attributed to PIF's influence on macrophage differentiation and function. PIF demonstrated a regulatory effect on irradiated macrophages and on classically activated M1 macrophages, reducing inflammatory gene expression (*iNOS, Cox2*), promoting protective (*Arg1*) gene expression and inducing pro-tolerance cytokine secretion. Notably, synthetic PIF is stable for long-term field use. Overall, clinical investigation of PIF for comprehensive ARS protection is warranted.

## INTRODUCTION

Acute radiation syndrome (ARS) is caused by high-dose whole or partial body radiation exposure [1, 2]. ARS may cause complete destruction of the bone marrow (BM), damage the mucosal barrier and crypts of the gastrointestinal (GI) tract, as well as skin burns and central nervous and cardiovascular system injury leading to irreversible neurological and cardiovascular damage and ultimately, death. In addition, the exposure to radiation can also increase the risk for development of cancer [3].

Radiation is particularly harmful to cells with rapid turnover such as those of the hematopoietic system and the gastrointestinal mucosa [2]. These injuries are associated with massive systemic inflammation and impaired immunity [2, 4–7].

Faced with the complexity of ionizing radiation induced injury, conventional ARS management lacks effective and comprehensive countermeasures. The current standard of care includes blood transfusion, fluid and electrolyte administration, antibiotic and antiviral therapy. Following low grade radiation, patients with cytopenia receive granulocyte colony-stimulating factor or granulocyte macrophage colony-stimulating factor to induce repopulation of the immune system from residual hematopoietic progenitor cells [4, 5, 7–9]. Non-responders and patients exposed to lethal radiation require hematopoietic stem-cell transplantation (HSCT) [5, 7, 9]. Such transplantation frequently leads to deleterious graft vs. host disease (GVHD) [10]. Not surprisingly, placental stem cells, with reduced antigenicity, were tested for ARS therapy [11]. However, using human-derived cells raises concerns with respect to purity, preservation quality, appropriate availability, and ultimate efficacy. Several other countermeasures have been examined but they are not currently approved for clinical use [12–16].

Ideally, ARS management should comprehensively cover all levels of exposure to irradiation (lethal or sub-lethal) both locally (colon, etc.) and systemically (i.e. hematopoietic, cytokines).

Pregnancy presents a unique immune environment wherein the embryo, a semi-allogeneic or allogeneic graft, is not rejected and does not lead to a graft vs. host response. Instead, pregnancy actually achieves ideal immune and transplant regulation. Therefore, the attributes of pregnancy could provide valuable guidance in the identification of effective and safe immuno-modulatory molecules. PreImplantation Factor (PIF) is an embryo-secreted 15–amino acid peptide which has an essential role in promoting embryo implantation and placental engraftment [17–23]. PIF presence in embryo culture media, placental and maternal circulation, is associated with favorable pregnancy outcome [24, 25] whereas its absence correlates with lack of viability. Synthetic PIF maintains its immunoregulatory properties in non-pregnant clinically-relevant models [20, 26–28]. PIF is effective against hypoxic brain damage plus an array of autoimmunity models, including those of neurodegenerative diseases, juvenile diabetes and atherosclerosis [29–34]. Importantly, PIF prevented deleterious semi- allogeneic as well as allogeneic murine GVHD following irradiation conditioning and preserved the beneficial Graft versus Leukemia (GVL) effect [10]. The protective effect was correlated with decreased levels of pro-inflammatory cytokines, both locally in target organs, and systemically.

Important mechanistic effects can explain the observed PIF induced protection in preclinical models. PIF directly regulates immune cell function by acting on macrophages and activated T cell proliferation, leading to a cytokine Th2 bias, as well as regulating NK cell activity [26, 28, 35]. In murine macrophages, PIF increased *B7h1* (ligated to PD-1 on T-cells) and decreased LPS-induced *Nos2* gene (*iNOS,* nitric oxide synthase) expression [10]. In human immune cells PIF targets protein-di-isomerase/thioredoxin (PDI/T) and heat shock proteins (HSPs) to reduce oxidative stress and protein misfolding [31, 32, 36]. PIF acts as a competitive inhibitor of cortisone binding to Kv1.3b where it reduces K+ ions release acting as a benign steroid [37]. Together, these *in vitro* and preclinical *in-vivo* results attest to PIF's potential as a countermeasure for ARS.

Herein, we examine PIF's potential as a treatment for ARS. PIF is used as a single therapy, without antibiotics, to provide mechanistic insight. Clinically relevant ARS scenarios were investigated following lethal and sub-lethal total body irradiation in murine models. PIF's effect on hematologic recovery when used alone, when combined with BMT, and when used for graft pre-conditioning was investigated. PIF's direct effect on cultured BM was determined. PIF's effect on systemic inflammation, local GI tract pathology, and global gene expression was tested.

We report herein that PIF offers comprehensive protection against ARS in the murine model and warrants clinical testing in humans.

## RESULTS

### PIF protects against mortality and restores hematopoiesis after lethal irradiation

**Figure 1:**
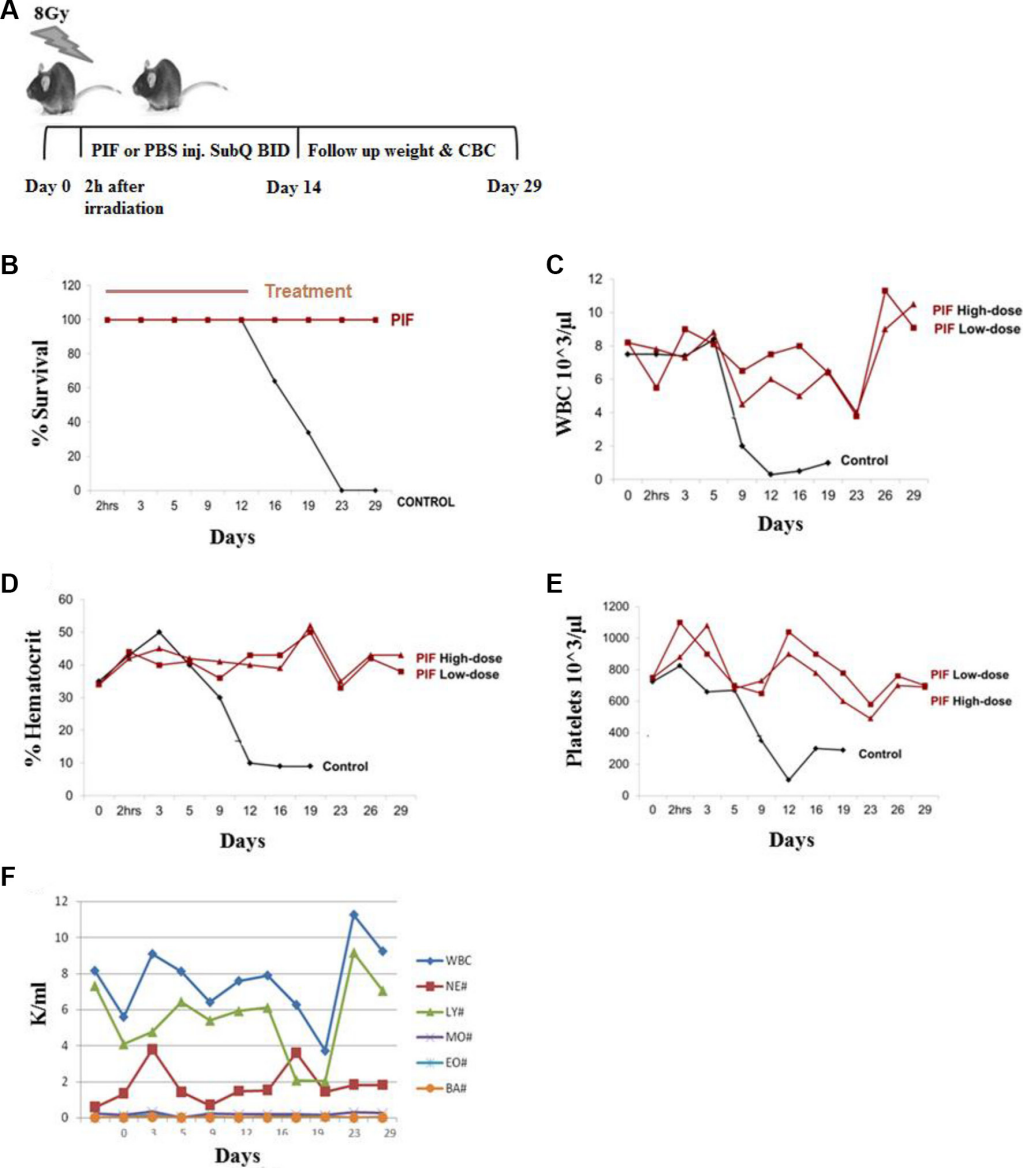
PIF enhances hematopoiesis after lethal irradiation Mice were irradiated with 8 Gy. Two doses of PIF treatment, 0.75 mg/kg (low dose) or 1.25 mg/kg (high dose), or PBS was administrated SQ 2x/day for 14 days starting 2h post irradiation. The protocol of the experiment is described in (**A**). Survival rate was monitored during 29 days post irradiation. The difference between the groups is significant. (*p* < 0.0001) (**B**). Blood count was compared: WBC (**C**), hematocrit (**D**), platelets (**E**) and different cell populations (**F**). No differences were found between pre-treatment and end of the experiments with respect to any hematologic index.

Early diagnosis and intervention are critical for the survival of patients who have been exposed to a lethal dose of ionizing radiation. To determine whether PIF is effective for such rapid intervention we used C57BL/6 mice, a relatively radio-resistant strain [38]. PIF treatment was initiated 2 h after whole body lethal irradiation (8 Gy) and its effect on survival and long-term hematopoiesis was studied. Female mice (C57BL/6, *n* = 18 per group) were treated with low-dose (0.75 mg/kg), high-dose PIF (1.25 mg/kg), or PBS control twice daily for 14 days, followed by 14 days of post-therapy monitoring (Figure 1A). Both PIF treatment groups had 100% survival until day 29, when they were sacrificed. In contrast, all the control mice (*n* = 14) developed ARS and had died by day 23 (0% survival) *p* < 0.0001 (Figure 1B). No significant differences were found between the two PIF doses tested. Figure 1C shows that mean white blood count (WBC) count was preserved on day 12, in both PIF-treated groups, as compared with the control group, which had a very low WBC count by that time. Remarkably, by day 29, WBC counts were restored to normal levels in both low and high-dose PIF treated mice. PIF also maintained the hematocrit levels throughout the experiment. In contrast, the control mice exhibited hematocrit levels of less than 10% by day 12 of the study (Figure 1D). Moreover, platelet counts in PIF treated mice remained high throughout the experiment. In contrast, in the PBS control group, platelet counts rapidly declined until day 12 and remained very low until demise of all mice. (Figure 1E).

PIF's effect on the recovery of different WBC cell populations following lethal irradiation and low dose PIF administration is demonstrated in Figure 1F. The lymphocyte time course resembles the global WBC. Reciprocal effects were seen between neutrophil and lymphocyte counts, namely a decrease in lymphocytes was associated with an increase in neutrophils, while monocyte, eosinophil and basophil levels remained practically unchanged.

Notably, similar results were obtained with male C57BL/6 mice (Supplementary Figure S1). Thus, prompt PIF administration as single therapy following lethal radiation prevents mortality and maintains hematopoiesis after therapy. The attached video shows PIF treated mouse recovery after lethal ARS (VIDEO Supplement).

### PIF treatment after lethal irradiation followed by semi-allogeneic bone marrow transplantation promotes transplant engraftment

Optimally, ARS therapy should be administered shortly after lethal irradiation (for prophylaxis) as above. However, therapy is frequently delayed due to inaccessibility and subsequently HSCT may be required. Since the donor must be available on short notice, haplo-identical allogeneic transplantation may be the only option. It was previously reported that PIF prevents GVHD development (skin ulceration, hepatitis and colon ulceration) after allogeneic bone marrow transplantation (BMT) [10, 39]. Whether PIF also promoted hematopoietic engraftment to restore immune profile critical for survival had not been determined.

**Figure 2:**
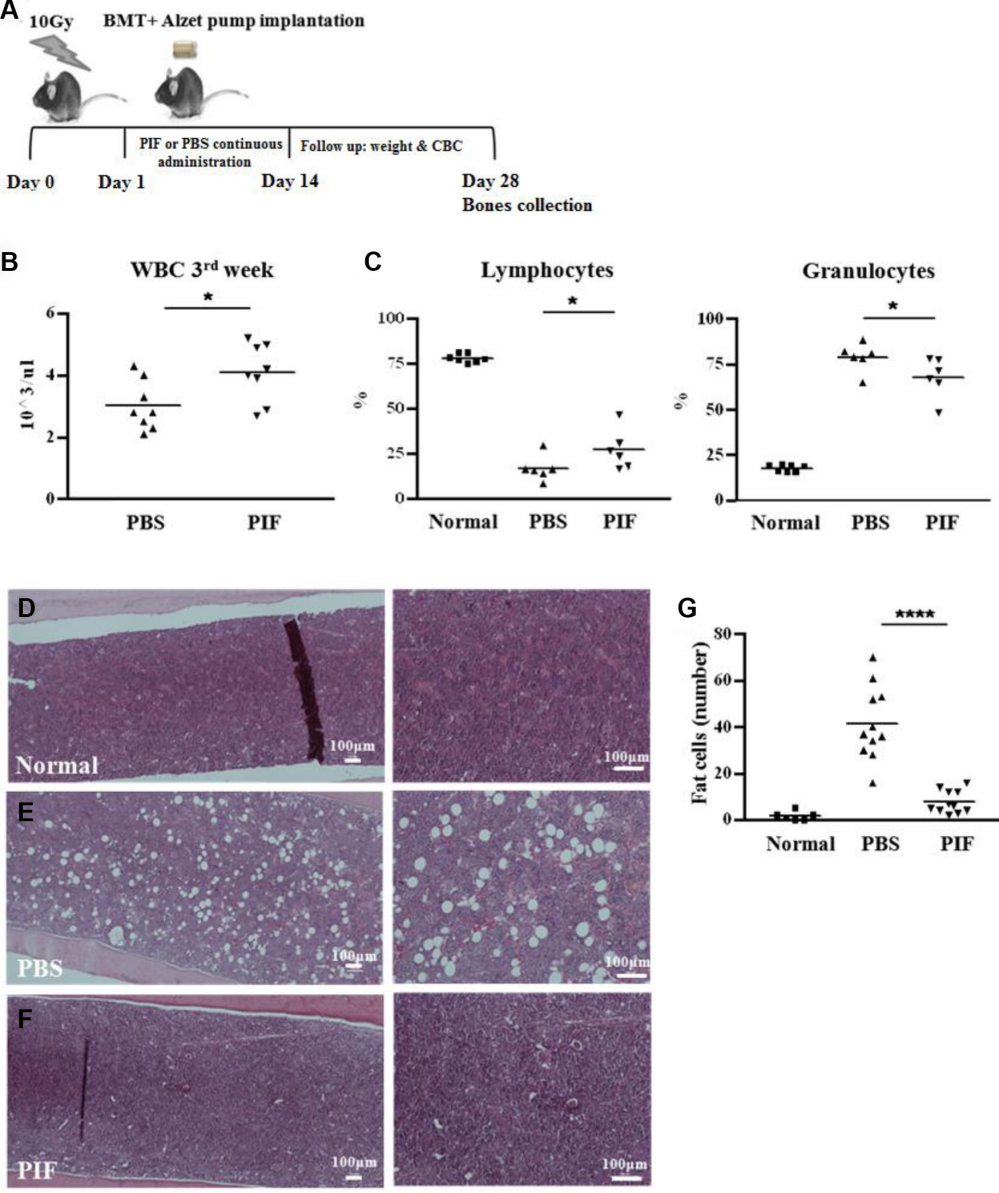
PIF improves hematopoiesis after lethal irradiation and semi-allogeneic BMT Mice were irradiated with 10 Gy followed by semi-allogeneic BMT. 1 mg/kg/day PIF or PBS were administered continuously for 2 w starting 24 h after irradiation. The protocol of the experiments is described in (**A**). WBC count 3 w after irradiation and transplantation (**B**). The percentage of lymphocytes and granulocytes 3 w post irradiation (**C**). Histological examination of the femur for cellularity level of BM in normal (**D**), PBS (**E**) and PIF treated mice (**F**). Fat cell number in 0.75 mm^2^ section of femur BM is a summary of 2 independent experiments (**G**). *p < 0.05, ****p < 0.001.

The present study therefore examined PIF's effect on hematologic recovery after lethal total body irradiation followed by semi-allogeneic transplantation (a model for haplo-identical transplant). In order to mimic a clinical scenario where access to care is not immediately available after exposure, PIF administration was delayed 24 h post exposure. F1 (C57BL/6 xBalb/c) mice were exposed to 10 Gy total body irradiation. On the next day the mice were transplanted with C57BL/6 BM by intravenous (IV) administration. PIF (1 mg/kg/day) or PBS was administered continuously starting at 24 h post-irradiation, for 2 weeks, using an Alzet osmotic pump (Figure 2A). Clinical condition and hematologic recovery were monitored for 4 weeks post-irradiation. It was observed that PIF-treated, compared to PBS-treated, mice showed significantly increased recovery of total systemic WBCs at 3 weeks post-transplantation, (Figure 2B). In addition, PIF treatment also improved the lymphocyte/granulocyte ratio when compared to PBS control mice (Figure 2C). At 4 weeks after transplant femoral bone histology was performed. Representative histologic images of femoral bone marrow of normal, PBS and PIF treated mice are presented in Figure 2D–2F, respectively. The fat cell number (index of the bone marrow reservoir) in the PIF treated group was significantly lower than that of the PBS treated mice (Figure 2G), demonstrating improved rehabilitation. Remarkably, fat cell number following PIF treatment was not significantly different from that of normal mice. Thus PIF, after lethal irradiation and BMT, rapidly restores both the circulating and the bone marrow hematopoietic reservoir.

### Transplantation of PIF preconditioned allogeneic bone marrow enhances hematologic reconstitution following lethal irradiation, without additional therapy

PIF's effect on the immune response was demonstrated in several previous publications [10, 26–28, 31, 32]. The above data documented PIF's ability to promote hematologic recovery following BMT. Whether PIF's effect is direct or indirect on bone marrow (BM) cells is unknown.

**Figure 3:**
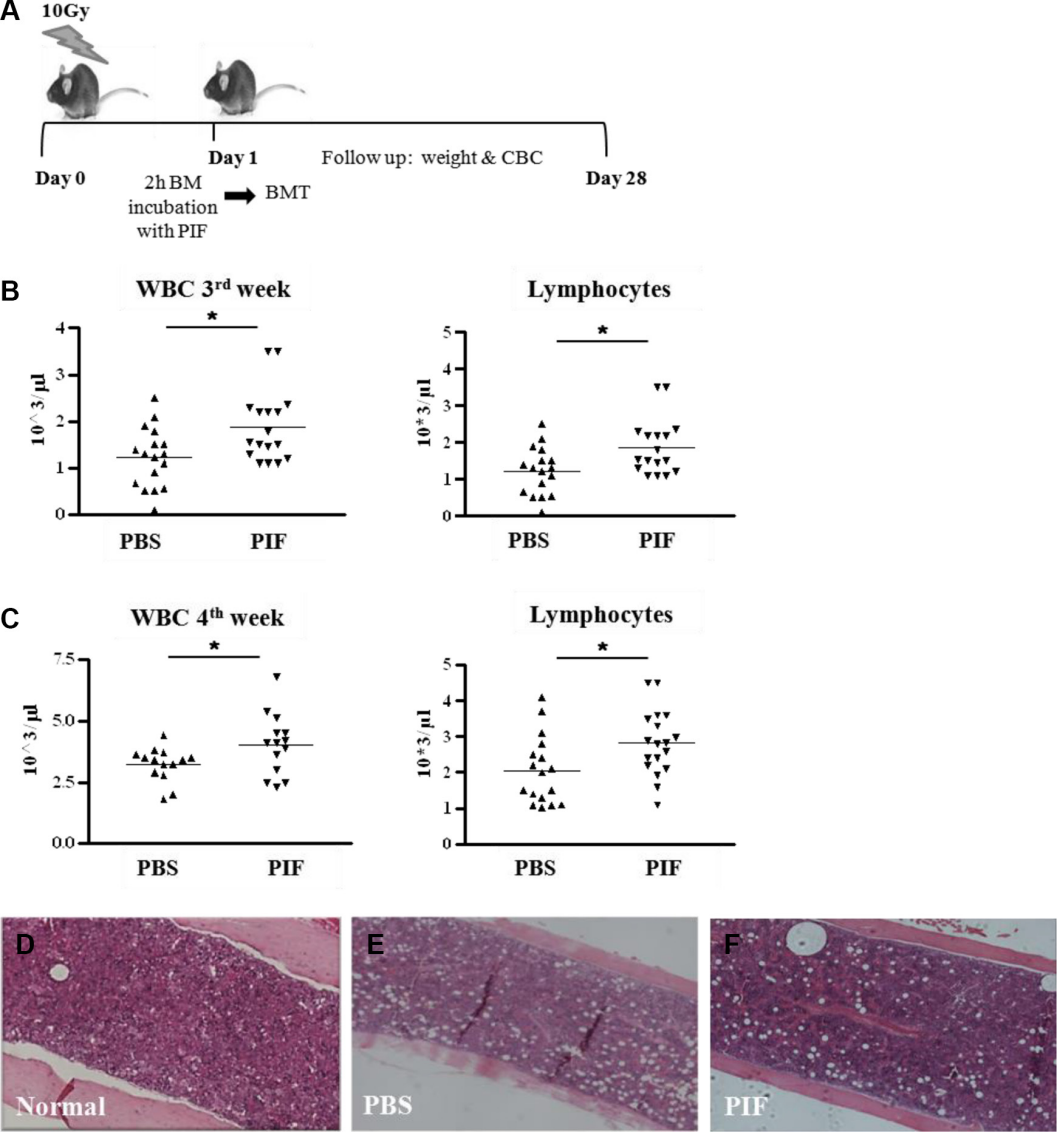
PIF pretreated BM enhances hematologic recovery after lethal irradiation and allogeneic BMT Donor BM cells were incubated with PIF for 2 h prior to transplantation Mice were irradiated with 10 Gy followed by allogeneic BMT with the pretreated BM graft. No additional treatment was given to the mice. The protocol of the experiments is described in (**A**). WBC and lymphocyte counts at 3 w (**B**) and 4 w (**C**) after irradiation and transplantation. Histological examination of the femur for cellularity level of the BM in normal (**D**), PBS (**E**) and PIF treated mice (**F**). Results represent 3 independent experiments. *p < 0.05.

To address this question, donor BM cells were pre-incubated with PIF in culture for 2 h. The cells were then washed prior to transplant. Recipient mice were lethally irradiated (10 Gy) and 24 h later the pre-conditioned allogeneic BM was transplanted. No additional treatment was administered throughout the 4 week monitoring period. The experimental protocol and plan are sketched in Figure 3A.

Although the BM was incubated with PIF for only 2 h prior to transplantation, its pre-conditioning significantly improved total WBC count, and, particularly, lymphocyte count, at 3 and 4 weeks post-transplant (Figure 3B and 3C, respectively). Improved engraftment was coupled with improved femoral bone marrow cellularity at 4 weeks (Figure 3D–3F). Thus, PIF pre-incubation alone leads to effective engraftment of BM cells without requiring further therapy post-transplant; this indicates a direct effect of PIF on the grafted cells.

### PIF exerts a direct effect on BM cells function *in vitro*

**Figure 4:**
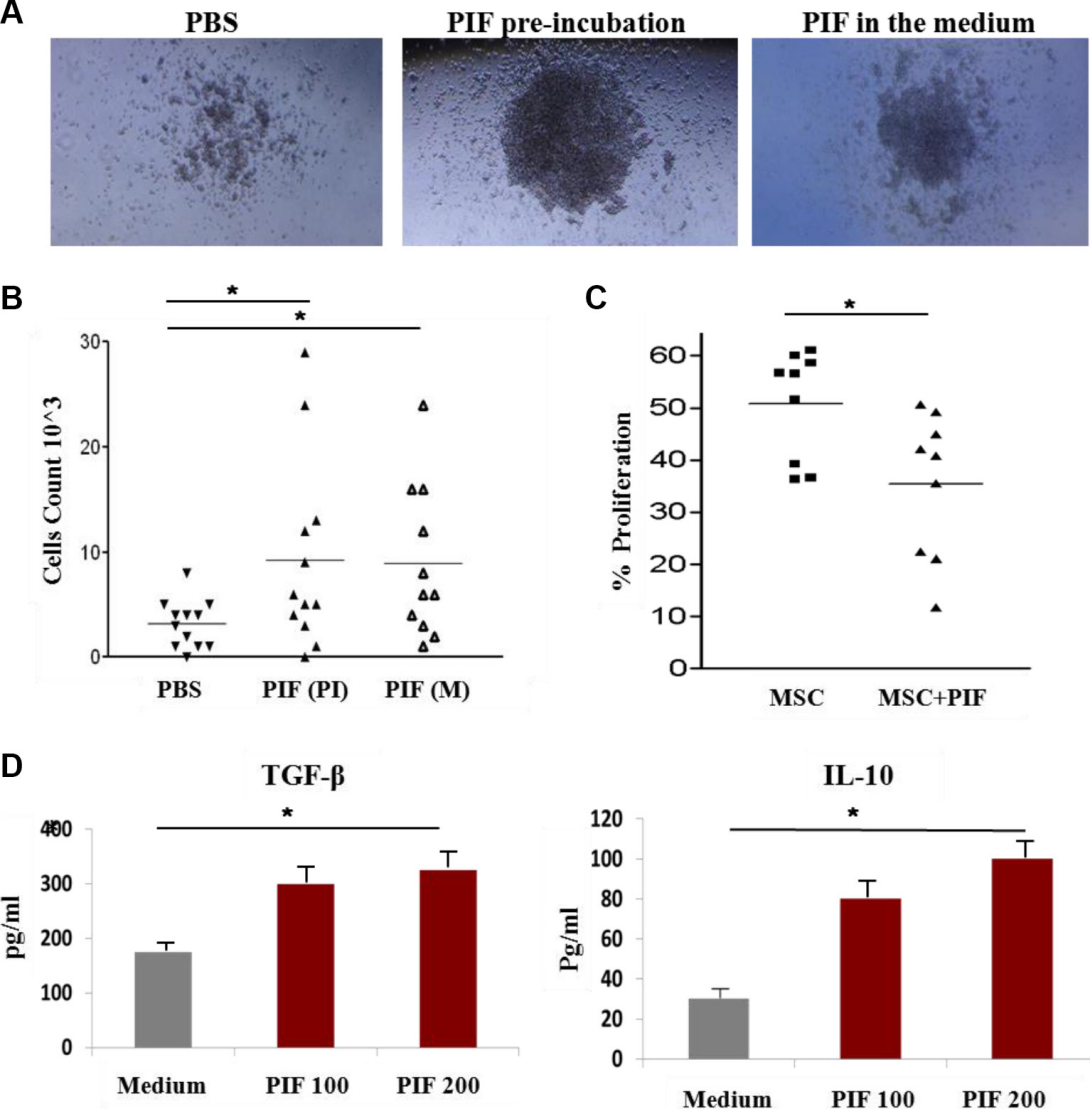
PIF improves pre-B cell differentiation from BM and enhances immunoregulatory function of MSC 2 h PIF pre-treated BM cells (middle), BM cells that were differentiated in the presence of PIF in the medium (right), and control PBS treated cells (left) were differentiated to pre-B cells in MetoCult M3630 differentiation medium (**A** and **B**). Pictures of representative colonies are represented in A and number of cells/well from 3 independent experiments in B. Inhibition of activated murine splenocyte proliferation by MSC and 2 h PIF pre-treated MSC, CFSE experiment (**C**). PIF effect on cytokine secretion from MSC, ELISA (**D**). Results represent 3 independent experiments. *p < 0.05.

To elucidate PIF's direct regulation of BM cells, its effect *in-vitro* was examined. First, we used MethoCult M3630 medium to differentiate pre-B cells from C57BL/6 mice BM progenitor/stem cells. In this experiment, 2 h PIF pre-incubated BM cells were compared with BM cells cultured in the presence of PIF or PBS (control) within the media (Figure 4A and 4B). The colonies formed were photographed and then the cells were harvested and counted. Following PIF exposure, with either pre-incubated cells or with cells cultured with PIF, larger colonies of pre-B cells were present (Figure 4A) and significantly higher B-cell counts were observed as well. (Figure 4B).

Another important cell population of BM are mesenchymal stromal cells (MSCs). These are multipotent progenitor cells which exert powerful anti-inflammatory and immuno-suppressive functions. Since we have already demonstrated PIF's immune modulatory properties in a variety of cells, its effects on the regulatory function of MSCs derived from C57BL/6 mice was next examined. MSCs were preconditioned with PIF for 2 h, washed, and then co-cultured with CFSE stained C57BL/6 splenocytes, activated with anti-CD3-antibody. The percentage of proliferating cells, as compared to activated splenocytes without MSCs, was determined by FACS analysis. The PIF-treated MSCs exerted a significant suppressive effect on splenocyte proliferation (65% inhibition) as compared to that of PBS control cells (50% inhibition) (Figure 4C). PIF added to the MSC culture also promoted secretion of pro-tolerance TGFβ and IL10 cytokines, both in a dose-dependent manner (Figure 4D).

These data demonstrate that PIF exerts a dual effect on transplanted cells, by promoting differentiation and by immune regulation.

### PIF alone promotes hematologic recovery at 24 h following sub-lethal irradiation dose

**Figure 5:**
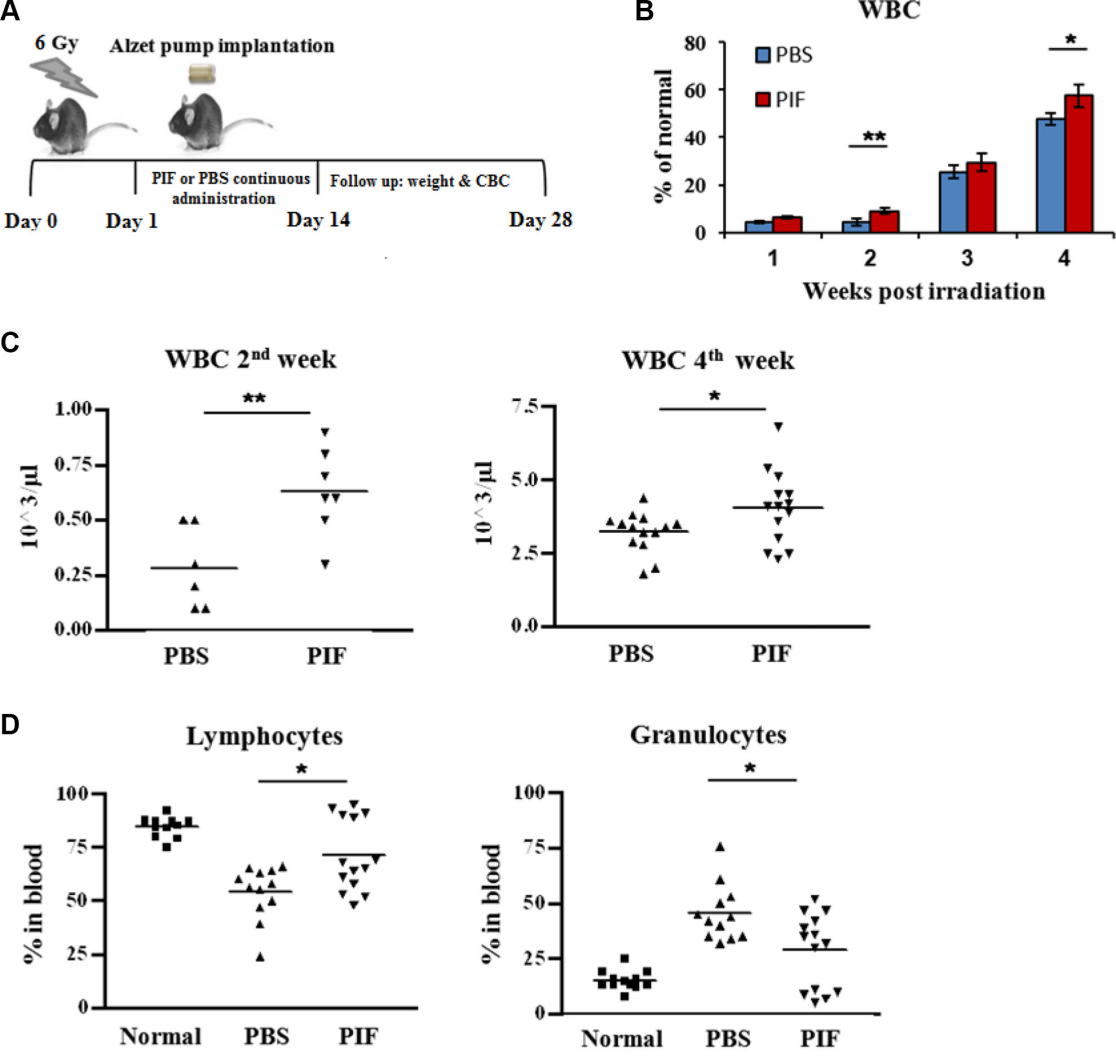
PIF enhances hematologic recovery after sub-lethal irradiation Mice were irradiated with 6 Gy. 1 mg/kg/day PIF or PBS was administrated continuously for 2 w starting 24 h after irradiation. The protocol of the experiments is described in (**A**). Follow-up of WBC reconstitution of the irradiated mice (**B**). WBC counts 2 and 4 w post irradiation (**C**). The percentage of lymphocytes and granulocytes 4 week post irradiation (**D**). Results represent 2–3 independent experiments. *p < 0.05, **p < 0.01.

The data above showed that early PIF administration protects against lethal ARS and is also effective after 24 h when combined with BMT. Next, the efficacy of PIF as a sole agent administered at 24 h after sub-lethal irradiation was examined. PIF (1 mg/kg/day) or PBS (control) were administered continuously (0.25 ml/h) for 2 weeks, starting at 24 h after 6 Gy irradiation. The radiation dose was chosen based on a radiation survival curve (Supplementary Figure S2). The clinical status and hematologic recovery were monitored for 4 weeks after irradiation. The experimental protocol and plan are sketched in Figure 5A. PIF aided in rapid recovery and significantly improved the reconstitution of circulating WBCs in comparison with PBS treated mice (Figure 5B). Two weeks post-irradiation, the mean WBC count was 600 cells/μl in PIF-treated mice as opposed to 250 cells/μl in PBS treated mice, reflecting improved recovery. The same trend was also evident at 4 weeks post-irradiation (Figure 5C). Moreover, at 2 weeks after its administration, PIF normalized the lymphocyte/granulocyte ratio by increasing lymphocyte percentage while reducing granulocyte percentage (Figure 5D). In contrast, in PBS treated mice, an elevated granulocyte percentage was noted, reflecting an enhanced inflammatory response. Thus, PIF treatment restores the immune profile after sub-lethal irradiation.

### PIF reduces circulating pro-inflammatory cytokine levels after sub-lethal irradiation

**Figure 6:**
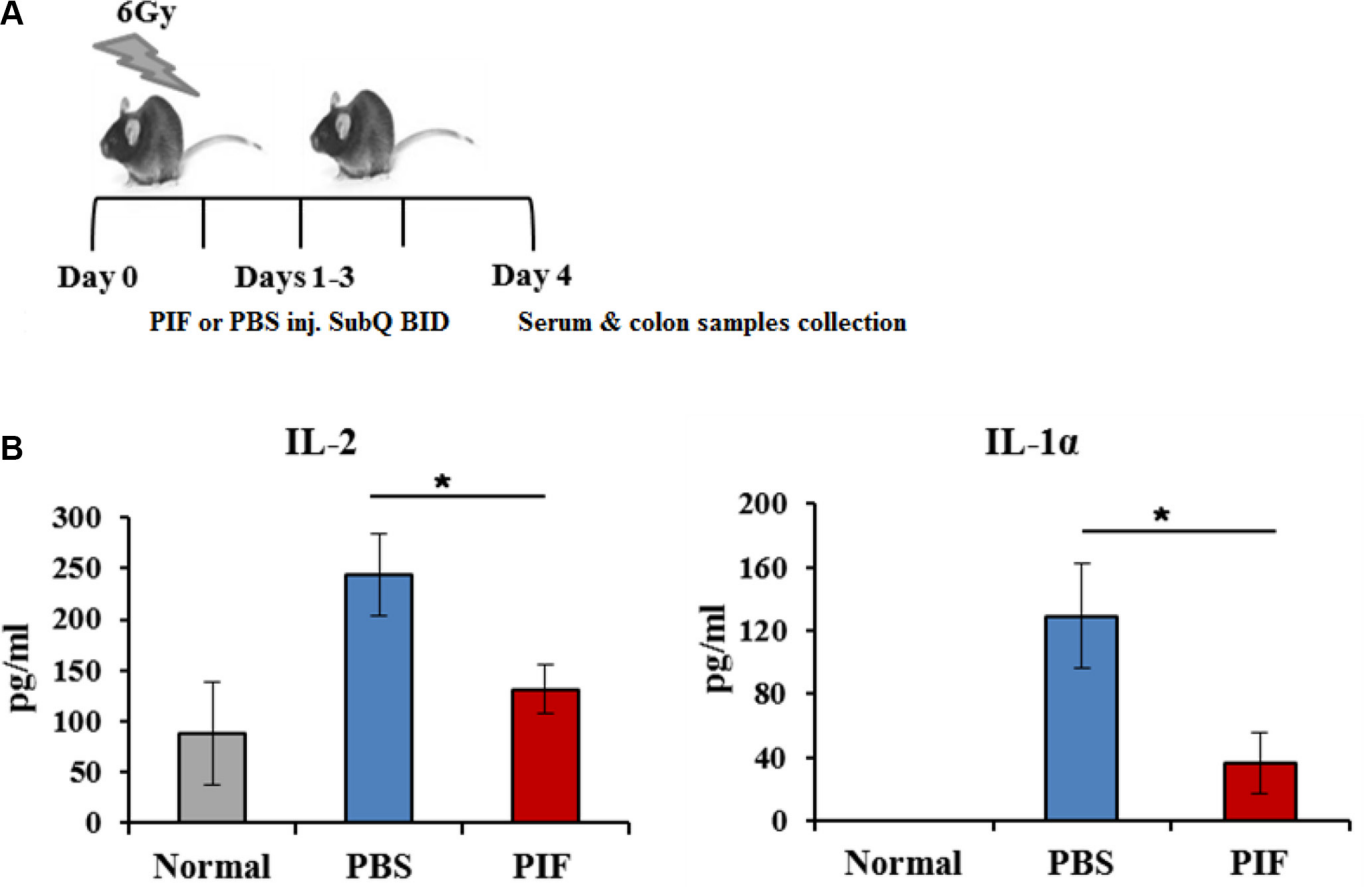
PIF reduces systemic inflammation after sub-lethal irradiation Mice were irradiated with 6 Gy. 0.75 mg/kg PIF or PBS were administered SQ 2x/day for 3 days, starting at 24 h after irradiation. The protocol of the experiments is described in (**A**). Levels of IL-1α and IL-2 in the serum of experimental mice were measured by FlowCytomix Multiplex kit (**B**). *p < 0.05

Inflammation and its associated circulating inflammatory cytokines contribute to ARS pathology. The effect of PIF on systemic inflammatory response was therefore examined. 24 h following 6 Gy total body irradiation, subcutaneous 0.75 mg/kg PIF or PBS was administered twice daily for three days (Figure 6A). Serum samples were collected for cytokine measurement.

Circulating levels of the pro-inflammatory cytokines, IL1α and IL2, were significantly decreased in PIF treated mice (Figure 6B). The effects on other cytokine levels (such as TNFα, GMCSF, IL10, IL4, and IL17) were below the limits of detection. These results imply that PIF administration after sub-lethal irradiation is effective in reducing systemic inflammation.

### PIF promotes regulatory macrophage differentiation

PIF's immune-modulatory effect on monocytes was previously demonstrated [10, 27, 40]. Activated macrophages secrete pro-inflammatory cytokines which can then contribute to ARS pathology, as demonstrated above (Figure 6B). To test the direct effect of PIF on activated macrophages, we examined PIF's protection against radiation-induced damage in macrophage culture.

**Figure 7:**
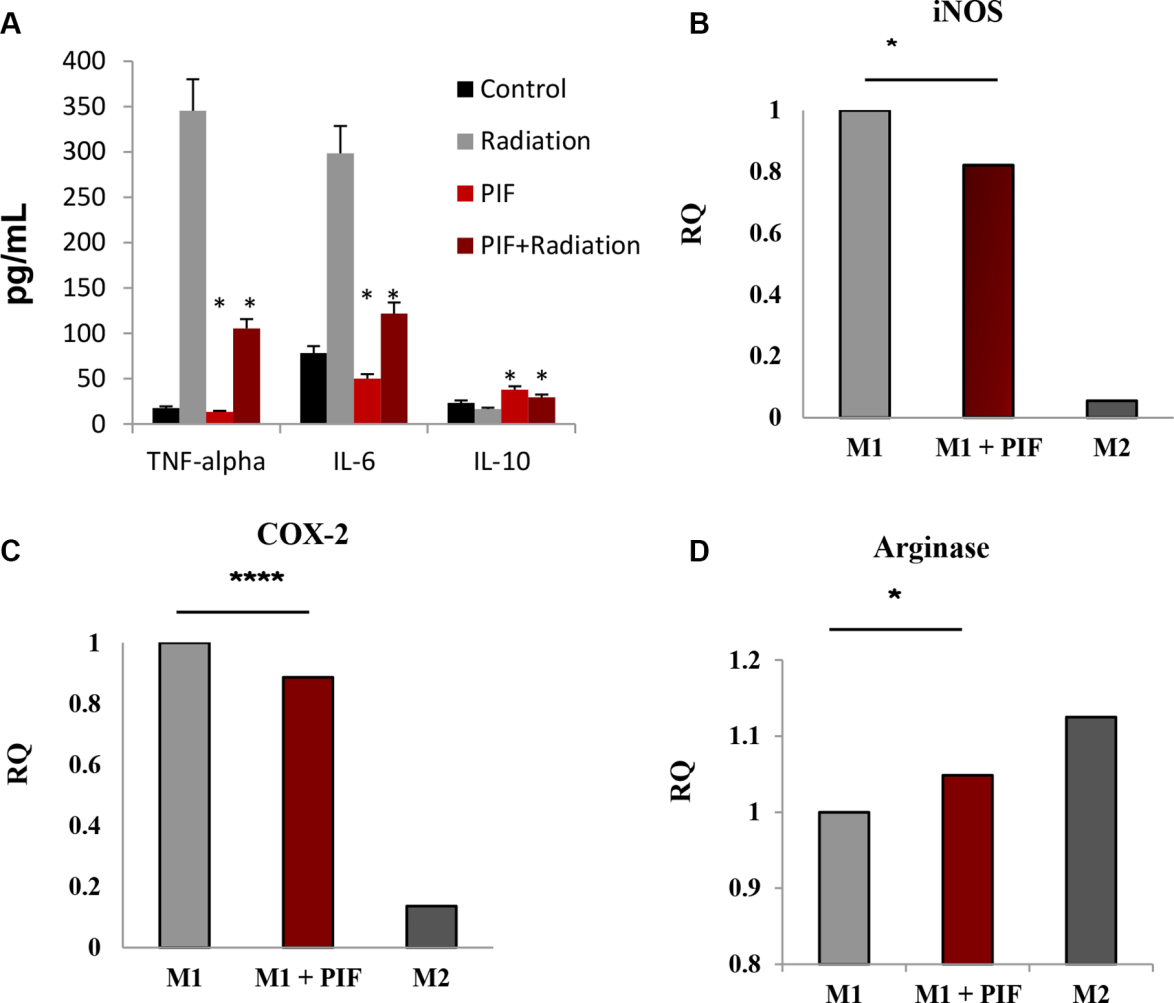
PIF promotes TH2/TH1 bias following irradiation and shifts M1 macrophage differentiation to M2-like phenotype (**A**) The RAW 264.7 macrophage cell line was employed in this study. The cells were irradiated (5 Gy) in the presence and absence of PIF (200 nM)- PIF reduced TNF- and IL6 while promoting IL10 secretion. Representative of 3 independent experiments. *p < 0.01. (**B**) Peritoneal macrophages were cultured with GM-CSF (10 ng/ml) and LPS (10 ng/ml) for M1 differentiation or with M-CSF (10 ng/ml) and IL-4 (10 ng/ml) for M2 differentiation for 20 h in the presence/absence of sPIF. qPCR analysis of iNOS (A) COX-2 (**B**) and Arginase (**C**) mRNA expression of the differentiated cells. % of M1 macrophages gMFI of F480 (**D**) and CD11b (E) by FACS analysis. Results represent 5–6 independent experiments. *p < 0.05, **p < 0.01 ****p < 0.001.

Following 5 Gy irradiation of RAW 264.7 macrophages, 200 nM PIF was added to the culture and its effect on the cytokine profile was determined. We focused on TNFα, IL-6, and IL-10 cytokines since they have been previously demonstrated to be affected by ionizing radiation [41]. PIF reduced TNF-α and IL-6 pro-inflammatory cytokine secretion while promoting IL-10 regulatory cytokine secretion. (Figure 7A). RT-PCR of the irradiated macrophages confirmed this inhibitory effect on TNF-α expression (data not shown).

Macrophages, as key mediators of the immune response, can differentiate into classically activated inflammatory macrophages (M1) or into alternative activated regulatory macrophages (M2). GM-CSF is reported to increase oxidative stress gene expression leading to pro-inflammatory macrophage M1 polarization. [42] We have previously demonstrated that PIF induces reduction of *iNOS* gene expression in the GVHD model [9]. Since *iNOS* is one of the inflammatory M1 macrophage hallmarks, we analyzed the influence of PIF on macrophage differentiation. Peritoneal macrophages were obtained from C57BL/6 mice and differentiated in culture towards M1/M2 phenotypes in the presence/absence of PIF (Supplementary Figure S3). In M1 macrophages PIF significantly decreased the levels both *iNOS* and *COX-2* gene expression (Figure 7B and 7C, respectively) and enhanced the expression of arginase, which is a marker of M2 regulatory macrophages (Figure 7D). Collectively, these results suggest that PIF regulates macrophage differentiation.

### PIF promotes colonic crypt and architecture recovery and regulates gene expression in the GI tract following sub-lethal irradiation

Since PIF reduced systemic inflammation by decreasing pro-inflammatory cytokines, we examined whether this was also coupled with local GI protection, a key element in early ARS, and where inflammation and injury are common [2, 6, 7]. PIF's ability to reduce colonic ulceration and liver inflammation in a murine GVHD model after lethal irradiation was previously noted [10]. Herein, mice were exposed to 6 Gy total body irradiation and then treated with subcutaneous 0.75 mg/kg PIF or PBS for 3 or 2 days, starting 24 or 48 h post-irradiation, respectively Figure 6A. GI tissue samples were harvested for analysis.

**Figure 8:**
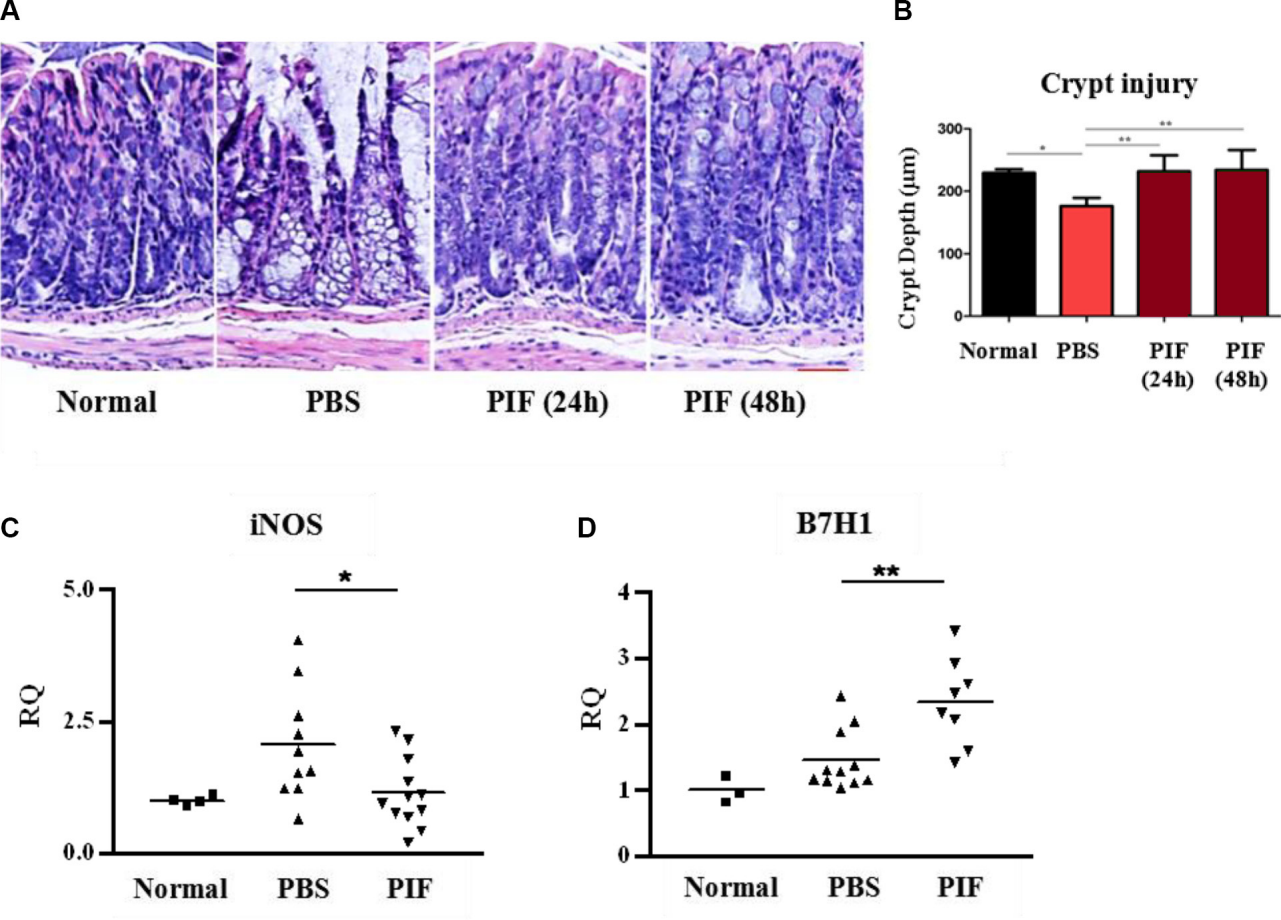
PIF treatment provides local colon protection after sub-lethal irradiation Mice were irradiated with 6 Gy. 24 or 48 h post irradiation mice were treated subcutaneously with 0.75 mg/kg PIF twice a day for 72 or 48 h, respectively. Histological examination of the colon at four days after irradiation (**A**). Statistical analysis of the colonic crypt depth of the different groups (**B**). qPCR analysis of iNOS (**C**) and B7H1 (**D**) mRNA expression in the colon. Results represent 2 independent experiments. *p < 0.05, **p < 0.01.

H&E staining showed that PIF significantly reduced colonic inflammation and restored colonic crypt morphology and colonic architecture even when PIF administration was delayed. In contrast, in PBS treated mice the colonic basal membrane became very thin, the colonic serosa was almost absent, and the intra-colonic architecture was completely disrupted (Figure 8A). Moreover, colonic crypt depth in PIF-treated irradiated mice was similar to that seen in normal mice and significantly different from that of the control mice (Figure 8B).

Colon genome analysis by real-time PCR also showed that PIF reduces *iNOS* gene expression as compared to the PBS control group (Figure 8C). Moreover, the expression of B7H1 in the colon of PIF treated mice was significantly up-regulated (Figure 8D). Therefore, PIF treatment reverses colonic inflammation even when administered up to 48 h after irradiation exposure.

**Figure 9:**
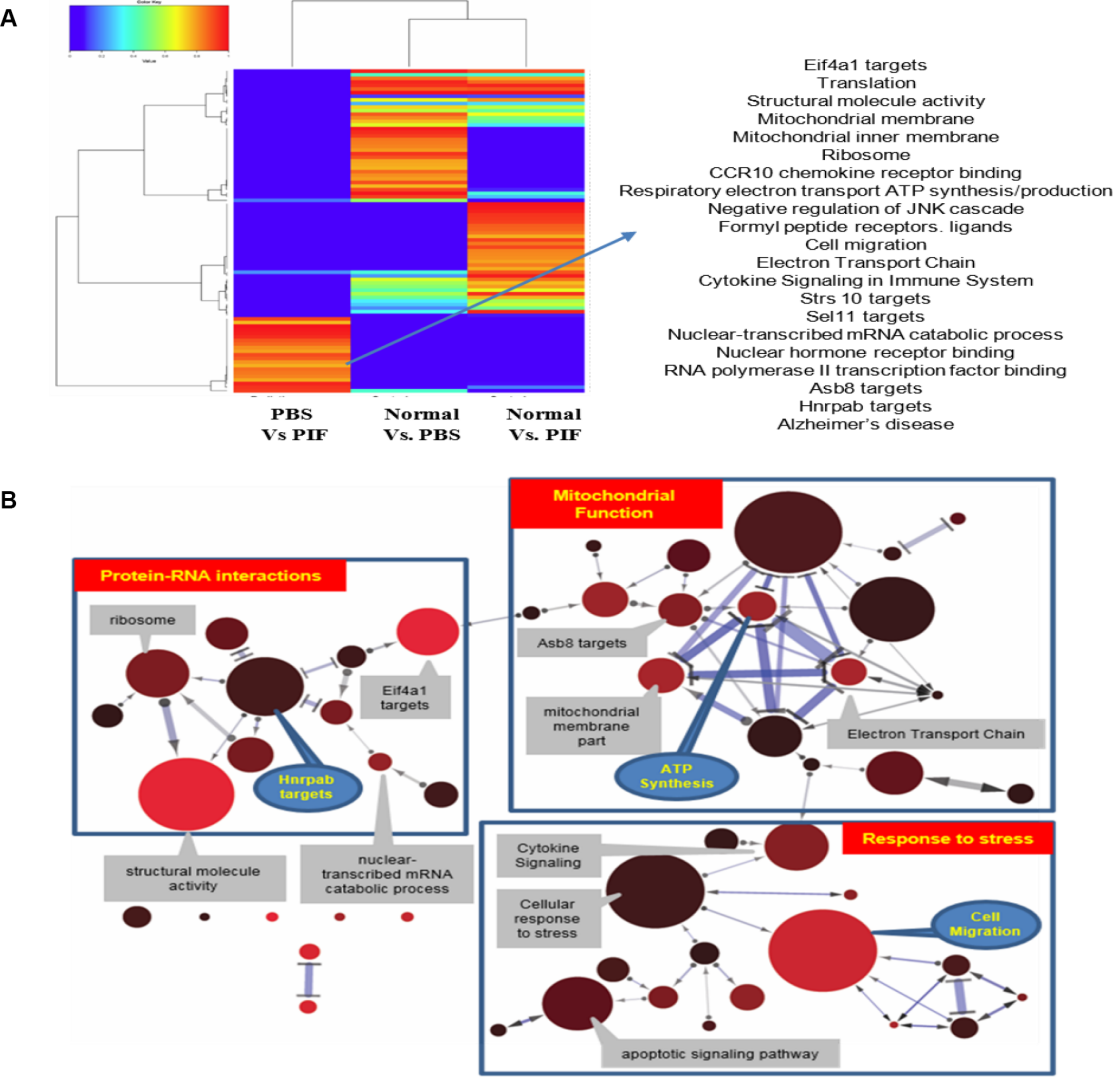
Heatmap analysis of the global GI genome Pathways analysis results of the RNA array data. The color scale indicates the relative rank of pathway in a given comparison, with 1.0 meaning top rank and 0.0 indicating a pathway that was not found significant at all (**A**). Gene sets for radiation exposure of the different treatments (PIF, PBS or normal mice). Every node represents a gene set. The size of a node reflects the number of genes in a gene set; node color reflects the significance (corrected *p*-value) with lower *p*-values with a red color (**B**). Edges denote overlap (i.e. 1 or more shared genes) between 2 gene sets. The width of an edge reflects the relative size of the intersection (Jaccard similarity) compared to both gene sets. Edges pointed one way denote relationships where subtracting 1 gene set from a second reduces the significance of the latter. In this case, the arrow points towards the more significant gene set. Bidirectional edges indicate subset relations; double lines indicate cases where the significance of both gene sets is solely due to the intersection between these.

The significant protective activity derived from PIF function was further examined by analyzing GI tract global gene expression using the Illumina array. A heatmap was created where the color scale indicated the relative rank of a pathway in a given comparison, with 1.0 meaning top rank and 0.0 meaning that a pathway was not significant. As shown in the heatmap analysis (Figure 9A), several affected pathways were detected when comparing the different sample groups (see Tables 1, 2 for ranking). The data were also used to draw an illustration of the network of genes affected by PIF treatment. This detailed network analysis of the relationship between the most important pathways (gene sets) for the irradiation plus PIF treatment versus irradiation treatment alone showed a clustering of gene sets into three main groups: genes involved in protein-RNA interactions, genes for mitochondrial functions related to oxidative pathways, and genes related to the response to cellular stress (Figure 9B). The top 20 genes, whose expression are up- or down-regulated by PIF in the GI tract as compared with PBS, are shown in Supplementary Table S1 and S2. The data generated confirm that the protection against ARS involves regulation of the inflammatory process whereas protection against ionizing radiation is coupled with metabolic and immune repair mechanisms.

**Table 1:** Ranking pathways involved in PIF action based on global GI gene analysis: comparison PIF to PBS – control

Control	PIF	Biological Process	Description
1	2.432692308	GO:0006979	response to oxidative stress
0.990384615	2.476934997	ITFP:Eif4a1	Eif4a1 targets
0.980769231	1.245192308	GO:0005198	structural molecule activity
0.971153846	1.242788462	GO:0016477	cell migration
0.961538462	1.240384615	GO:0035257	nuclear hormone receptor binding
0.951923077	1.237980769	ITFP:Lman1	Lman1 targets
0.942307692	1.235576923	GO:0031735	CCR10 chemokine receptor binding
0.932692308	1.233173077	WP295	Electron Transport Chain
0.923076923	1.230769231	GO:0044455	mitochondrial membrane part
0.913461538	1.228365385	5893258	Respiratory electron transport, ATP synthesis by chemiosmotic coupling
0.903846154	1.225961538	GO:0001085	RNA polymerase II transcription factor binding
0.894230769	1.223557692	ITFP:Sel1l	Sel1l targets
0.884615385	1.221153846	5893543	eNOS activation and regulation
0.875	1.21875	GO:0046329	negative regulation of JNK cascade

PIF effect on gene pathway ranking in global GI genome was determined and compared with PBS (vehicle) treated mice exposed to sub-lethal- 600 rads. Following sacrifice the tissue mRNA was extracted and ran on an Illumina chip. (*N* = 7/group) Data generated were analyzed, determining strength of association of a pathway affected by PIF. Further details are described in the method section.

**Table 2:** Specific GI genes affected in the different pathways—comparison of PIF to PBS treated mice

Response to Oxidative Stress		Hnrpa targets		Memnbrane Hiking		PSMD4 t		argets + EIF4A1 Targets		Translation		NR3cl Targets		R-SMAD bindings+		Nuclear Specks	Alzheimers	
+	−	+	−	+	−	+	−	+	−	+	−	+	−	+	−	-	+	−
APOA^4^	SRXN1	H2AF2	CDL20	HSPA8	SFN	PSMA7	E1F2S2	SCD1	H3FBA	RPS3	ETF1	MBNL2	SGPP1	FOS	ZC3H3	PRPFIQ	ATP5F1	APAF1
ADIPOQ	GPX2	AIMP^2^	G^3^BP()	CALM2	COPA	NHP2	PSMD7	E1P5A		RPL7a	RPN2	MBNL1	DROSHA	PP1MA	SMAD3	EP400	CALM2	PPP3A
CASP^6^	PML	RPS^3^	DPP^3^	AP1S1	AP161	POLR1D		PPPlca		RPS27a	(SEC61a2)	ITM2B	ACOX3		ZEB2	MBD1	COX7A21	ITPR3
GPX^4^	KDM6B	NHP2	GLMP	SAR1B	YNHAH	AK2	RPS7		SP5CL	RP524							ATP52	IDE
FOS	ATP7	POLR1D	SSR2	RPS27a	PRKAB1	TIMM8B		E1F5		Eig5	RPL31						NDUFA6	ATP2A3
NDUFA6	MAPK7	EiF3G	TLXNA		AP1B1	MRPL9		RPL18		EEF2	EiF4cBPl						SDHB	
	ATOX1	RP57	RNPS1		AP^3^B^1^	MTCH2				RPL18							NDUFS3	
		RPL18	SAE1		YWHAB					RPL13a							ATP5CL	
		RPL13a	ECT^3^		GBFL					Ei3g							LPL	
		RPLPO			COP2L												NDUFS4	
					PREB													
					HGS													
					GNS													
					GT5Z													

PIF effect on the individual genes of the global GI genome was determined and compared with PBS (vehicle) treated mice after 24 hours exposure to sub-lethal- 600 rads. Following sacrifice, the tissue mRNA was extracted and ran on an Illumina chip. (N = 7/group). Data generated were analyzed, *determining association of a given pathway with genes affected by PIF. (Heatmap analysis)- figure 8. Further details are described in the methods section.

### PIF is highly stable and fit for rapid administration

The utility of a drug is based on its availability and convenience of use in an adverse environment. To further establish PIF's durability, stability testing was carried out by using HPLC and mass spectrometric analysis of clinical grade synthetic PIF. After 8 weeks of storage at ambient temperature, PIF's clinical grade quality was maintained, making it suitable for field use (Supplementary Table S3). Thus PIF is suitable for prompt use in the field, where accidental or deliberate exposure to radiation may occur.

## DISCUSSION

ARS due to total body irradiation leads to local and systemic inflammation followed by long-term hematologic failure and organ damage. Despite ongoing efforts, current therapies are of limited efficacy and most often are associated with significant risks. There are no new approved drugs that would comprehensively address ARS. The major finding in this study is that PIF, administered as a single therapy, accomplishes this comprehensive ARS treatment capability, covering the spectrum of the disease.

ARS is a complex disease which can affect practically any organ in the body. The degree of radiation damage is related to dose, duration, whether exposure is total or targeted to only a selected organ, and it also importantly depends on the host's susceptibility. The aim of this study was to comprehensively cover possible ARS scenarios, both short and long-term consequences, and with a clear connotation for future clinical translation.

Endogenously secreted, PIF, due to its embryo-derived origin and function, plays an essential role in the maternal recognition of pregnancy that is, protecting the embryo/allograft against rejection while achieving maternal/host tolerance through immune modulation (but not immune suppression) [34]. Synthetic PIF administration was previously shown to duplicate the pregnancy-observed global immune and transplant regulatory effects of native PIF, and presents the qualities needed to address the ARS spectrum of disease. Observations in both *in vitro* and in clinically relevant preclinical models support the view that PIF therapy could be useful as a comprehensive local and systemic countermeasure against ARS. PIF was found to preserve local GI homeostasis, restore bone marrow cellularity and improve systemic hematopoiesis. This protective effect is further potentiated by a macrophage shift, from inflammatory to regulatory type, combined with reduced circulating pro-inflammatory cytokines.

PIF administration immediately after exposure to lethal radiation provides a rapid and comprehensive response to accidental (i.e. nuclear power plant) or medical (radiation oncology) exposure. Importantly, immediate PIF treatment (within 2 h of irradiation) protects against high-dose radiation mortality, preserving blood indices without any additional therapy. At no point was significant reduction in hematocrit or platelet count was noted, while WBC levels oscillated but did not reach immune suppressive levels. Comparable protective effects on both female and male mice, coupled with the normal endogenous exposure to PIF in all offspring throughout pregnancy, supports a gender independent clinical application potential for ARS therapy.

In cases of deliberate exposure, the treatment is delayed until victims are brought to medical care facilities. In both sub-lethal irradiation as well as in lethal irradiation circumstances followed by BMT, PIF was shown to impart an effective and comprehensive treatment for radiation injuries, even when administration was initiated 24–48 hrs post-irradiation. Currently HSCT is the mainstay for long-term management of lethal ARS when immediate intervention is not available. However, HSCT can be difficult to arrange on a short notice since donor-matching and availability is complex. Therefore, the ability to transplant without complete matching remains a major challenge. We have previously reported that following allogeneic BMT, PIF administration led to reduced morbidity and mortality [10]. Herein we demonstrated that PIF, through a short 2 hour preconditioning of the BM, improves hematopoietic recovery after semi-allogeneic BMT without further therapy, indicating a direct regulatory effect of PIF on BM cells. Such an approach may positively impact both ARS and HSCT management. Together with previously reported data demonstrating the beneficial effect of PIF treatment on GVHD [10], PIF treatment may offer a long-term solution for allogeneic BMT patients.

The direct effect of PIF on BM cells was also demonstrated *in vitro*, by improved differentiation of pre-B cells in culture, reduced adverse immune activation and enhanced pro-tolerance cytokine secretion of PIF treated MSCs. Thus, the mechanism of PIF protection in ARS probably includes both immune-modulatory effects (as demonstrated in several of our previous publications, [26, 27, 40] as well as in our present results), and direct effects on bone marrow cells.

Exposure to sub-lethal irradiation may cause severe chronic damage from secondary complications. Such exposure damages hematopoiesis, and causes both systemic as well as local inflammation. PIF increases the lymphocyte/neutrophil ratio after sub-lethal irradiation; this persisted weeks after stopping therapy, providing a long term therapeutic solution. The systemic inflammatory response is rapid, aggravating ARS by promoting pro-inflammatory cytokine increase. The PIF-induced reduction of circulating IL-2 and IL-1α to levels seen in healthy mice indicated lowered systemic inflammation. IL-2 is a prime stimulator of the adaptive immune response and its activation shortly after exposure would be clearly detrimental. IL-1α is a pro-inflammatory cytokine that is released by macrophages and acts in synergy to stimulate IL-2 secretion. Indeed, PIF enhanced pro-tolerance cytokine secretion in radiation treated macrophages, providing direct evidence for targeted protection. M1 macrophages are pro-inflammatory and have a central role in host defense against infection, while M2 macrophages are associated with responses to anti-inflammatory reactions and tissue remodeling. M1-M2 macrophages represent two distinct directions within the full spectrum of macrophage activation. We have previously demonstrated that PIF binds to monocytes leading to their differentiation to an immune-regulatory type [10]. Additionally, down-regulation of *iNOS*, gene expression, a M1 macrophage hallmark, was evident in target organs of PIF treated mice in both the current ARS model and in our previous GVHD model [10]. In this study, the addition of PIF to M1 macrophages reduced the M1 phenotype by decreasing the levels of *iNOS* and *COX-2* gene expression. In macrophages that express *iNOS*, the level of *COX-2* expression is an important indicator of the degree of inflammation [43]. Thus PIF-associated decreases in *iNOS* and *COX-2* expression correlate with decreased serum levels of IL1α and colonic *iNOS* gene expression, as observed in our *in-vivo* model.

The GI tract has significant metabolic functions beyond digestion and electrolyte balance. It keeps the local micro-organism flora in check, and also serves primordially as an immune organ. Localized GI inflammation leads to cytokine release which activates systemic immunity. Since the colon has a rapid cellular turnover it is specifically sensitive to apoptosis and reduction in local stem cell function as a result of radiation [2, 44]. ARS-induced GI tract damage can therefore severely impact patient prognosis, leading to diarrhea, dehydration, and infection due to the altered bowel flora and function. Therefore, in order for a given therapy to be effective, both local and systemic compartments have to be effectively controlled.

Remarkably, even at 48 hrs after sub-lethal irradiation and with only a 2 day administration, PIF protected against colonic inflammation and preserved normal colonic crypt depth and length values. By preserving basal membrane integrity PIF prevents diffusion of toxins, which may activate a systemic inflammatory response, from the colon. *B7h1* is a key molecule involved in protecting the colon against inflammation, a function demonstrated with *B7h1*-knock-out mice which develop severe chemically-induced colitis [45]. We previously demonstrated that PIF up-regulates *B7h1* expression in macrophages which may negatively regulate T cell activation through association with the PD-1 ligand [10]. Herein, the decrease in *iNOS* and Nosip gene expression in the GI tract, combined with increased expression of the *B7h1*gene promotes local immune protection. Therefore, PIF could be hypothesized to also be useful for treatment of colon inflammation or ulceration, unrelated to ARS therapy.

Global genome analysis, coupled with the heatmap analysis, provides a comprehensive insight into PIF protection of the GI tract. The PIF-induced effect was compared to both vehicle treated control and to healthy mice. One of the pivotal mechanisms to generate proteome diversity and complexity is alternative pre-mRNA splicing where aberrant splicing predisposes to chronic inflammation [44, 46]. The *Hnrpab* pathway was used also to demonstrate PIF-induced protection in neural and immune cells [30, 47]. In the current model of ARS, the mitochondrial gene cluster, which PIF affects, involves regulators of reactive oxygen radicals and intracellular Ca2+ signaling molecules. This effect is relevant in inflammation control [45, 48], and in PIF-induced promotion of neural cell survival [30, 47, 49–51]. Moreover, PIF was shown to regulate immune gene clusters and proteins [10, 31, 32]. Beyond the increase in the critical *B7H1* gene (colon), PIF also reduced *Panx1,* involved in inflammasome activation,* and *Cxcl13* expression,* which affects B-cell chemotaxis to Peyer patches. PIF also increased *Olfactomedin4, Csp6* and *Lysozyme1* expression. *Casp6* acts as executioner caspase and is likely to prevent GI cell apoptosis, while the increased *Lysozyme1* expression can prevent infection and inflammation development. PIF may also influence the potential oncogenic propensity of the GI tract by regulating *Ccnd*, a cyclin dependent protein serine kinase [52, 53]. The role of all genes regulated by PIF is the basis of further investigation.

Several agents have been evaluated for ARS therapy following lethal and sub-lethal irradiation exposure. Current interventions are mainly supportive, or involve hematopoiesis inducing agents like GM-CSF and, as recently suggested, IL-12, P selectin antibody, MSCs, and TLR ligands [12–14]. Specifically MSC action appears to be dependent on reducing the IL-17 inducing signaling in the colon [15]. Recently high-dose intraperitoneal TP508 peptide injection protected against GI inflammation at 24 h post-lethal radiation, reducing mouse mortality by 30% and increasing survival from, on average 12.4 to 16.8 days. As the authors stated, for long-term survival, additional life-saving medical treatment (i.e. BMT) is needed and is to remain as part of the solution [16].

The representative murine model used in the present study demonstrates that PIF comprehensively addresses the spectrum of ARS clinical scenarios, from lethal irradiation to sub-lethal exposure, with immediate or delayed administration, and from acute to chronic manifestations of the disease. Even when early treatment is unavailable and BMT becomes inevitable, PIF treatment or PIF BM pre-conditioning potentially offer a novel long-term solution for lethal irradiation management. PIF therapy is shown to prevent systemic inflammation, to provide long-term hematopoietic reconstitution and to restore GI tract functionality.

Ease and accessibility of treatment is essential for effective ARS management. PIF is stable even after 8 weeks at ambient temperature and therefore is highly utilitarian for both military or civilian needs. In addition to ARS, PIF may be considered as a therapy in other radiation induced syndromes. For example, radiotherapy is an important component in breast cancer treatment as it reduces recurrence and improves overall survival. However, such treatment raises the risk of acute and chronic side effects including radiation pneumonitis (RP), an early inflammatory reaction that occurs four to twelve weeks after completion of thoracic irradiation [54]. Our current results support the efficacy of PIF in reducing local irradiation induced inflammation, which is relevant for RP patients as well as for patients suffering from other radiotherapy induced complications.

The efficacy of PIF observed in the current murine ARS model is valuable for translation to clinical testing. Importantly, PIF's safety was demonstrated. PIF treatment is currently being tested in an ongoing University-sponsored, FDA Fast-Track approved clinical trial for an autoimmune disease manifested in liver inflammation (NCT02239562). Therefore following this Phase I safety trial PIF could be also tested for the mitigation of radiation induced injury.

## MATERIALS AND METHODS

### Mice

The lethal 8 Gy ARS study was conducted under ethical conditions approved by the IACUC NIH OLAW (A4475-01), U.S. Dept. of Agriculture (USDA) and carried out by the BATTS Laboratory (Northridge CA). All the subsequent studies were done at the Hebrew University of Jerusalem (Israel) and approved by the Institutional Animal Welfare Committee (Israel). C57BL/6 mice (6–7 or 8–9 w) and F1 (C57BL/6xBalb/c) mice (10–11 week old female) were obtained from Jackson Labs (Maine) and Harlan Laboratories, Ltd. (Israel), respectively. All mice were kept in pathogen free cages and housed under specific pathogen-free (SPF) conditions with a twelve-hour light cycle. No antibiotics were administered.

### PIF synthesis and administration

Synthetic PIF (MVRIKPGSANKPSDD) was obtained from Biosynthesis, Lewisville, NJ USA. All peptides were purified to > 95% purity, documented by mass spectrometry before being tested.

PIF peptide was dissolved in Dulbecco's phosphate-buffered saline (PBS) with 0.2% Dimethyl sulfoxide (DMSO) (Biological Industries and SIGMA, respectively). 0.75 mg/kg or 1.25 mg/kg PIF was injected twice a day, SQ, for 14 days. For hematopoietic recovery studies in lethal irradiation+BMT and in sub-lethal studies, 1 mg/kg/day PIF or PBS was administered continuously (0.25 ml/h) starting one day post-irradiation and for 2 w,* using Alzet osmotic pumps (Model 1002, Durect Corp., CA), implanted SQ [10].

### ARS Models: PIF therapy without BMT

C57BL/6 mice underwent lethal whole-body irradiation by single exposure to 8 Gy (BATTS laboratory) or sub-lethal whole-body irradiation by single exposure to 6 Gy (Hadassah Hebrew U). The survival curve 6–10 Gy is shown (Supplement) All mice were irradiated with gamma radiation at a dose rate of 0.3 Gy/min. To minimize stress, no anesthesia was used enabling mice to move freely in the cage assuring uniform radiation exposure.

### PIF therapy with BMT

F1 mice underwent lethal whole-body irradiation by single exposure to 10 Gy (Hadassah Hebrew U). One day post-irradiation, BM mononuclear cells from C57BL/6 donor mice were collected by flushing the femur and tibia with PBS (Biological Industries). Mononuclear cells were isolated using Lymphoprep. 8 × 10^6^ BM cells were administered through the tail vein of irradiated recipient mice. In another set of experiments, BM cells were pre-incubated with PIF for 2 h before transplantation.

### Clinical examination and complete blood count (CBC)

Following radiation exposure, mice were monitored daily for weight loss, ruffled skin, and survival. Blood was collected, according to the protocols, from the retro-orbital region or from the tail (BATTS Laboratory or Hadassah Hebrew University, respectively) into Ethylenediaminetetraacetic acid (EDTA) coated capillary tubes. CBCs with differentials were performed using a validated BC-2800Vet Auto Hematology Analyzer (Mindray).

### B cell proliferation

BM cells obtained from C57BL/6 mice were incubated (2 h) with PIF or control prior to use. In some samples PIF was washed before culture. Cells were suspended in 200 ul IMDM (2% FBS) and then mixed with 2 ml of MethoCult M3630 (Stem Cell Technologies). The cells were then cultured (250 ul/well in 24 well plates) for 7 days.

### Mesenchymal stromal cell (MSC) experiments

Mouse MSCs were cultured from BM as previously described [55]. BM cells obtained from C57BL/6 mice were cultured in Mesencult basal medium supplemented with MSC stimulatory supplements (Stem Cell Technologies), at 37°C with 10% CO2. After two to three passages, the cells were harvested and CD11b+ cells were depleted by EasySep cell separation kit (Stem Cell Technologies). After two more weeks in culture the phenotype of the cells was determined using Flow Cytometry. For the experiments, MSCs were incubated (2 hrs) with PIF or control prior to use in co-culture with activated splenocytes. CFSE (Thermo Fischer Scientific) stained murine splenocytes were activated with anti-CD3 antibodies, (Pharmigen) for four days in co-culture with MSC (in a 1:50 ratio). Cell proliferation was analyzed using Flow Cytometry.

### MSC cytokine secretion

The levels of IL-10 and TGFβ in the supernatants from cultures of PIF/PBS treated mouse MSCs were analyzed by enzyme-linked immunosorbent assay (ELISA) kits according to the manufacturer's instructions (Thermo Fisher Scientific). The experiments were performed in triplicates to ensure reproducibility.

### Histological analysis

Colon and femur samples were obtained from mice and fixed in 4% neutral-buffered formalin. Bone samples were decalcified, embedded in paraffin, cut into 10-micron thick sections and stained with hematoxylin and eosin (H&E).

Colon samples were embedded in paraffin and then processed into 5 mm sections for H&E staining (Fisher Scientific). Crypt depth was analyzed by a BX51 microscope (Olympus) equipped with a digital camera and images acquired using a 10× objective. The images were analyzed using ImageJ software.

### Serum cytokine evaluation

Mouse serum was obtained from peripheral blood 96 h post-irradiation. Circulating cytokine levels were determined by Mouse Th1/Th2 10 plex FlowCytomix Multiplex kit according to the manufacturer's protocols (eBioscience).

### Macrophage cytokine secretion

The RAW 264.7 macrophage cell line was employed in this study. The cells were irradiated (5 Gy) in the presence and absence of PIF (200 nM). Cytokines TNF-, IL-6, and IL-10 secretion were analyzed at 48 h of culture using an ELISA kit and confirmed by PCR. (ThermoFisher Scientific).

### Macrophage isolation and differentiation

Peritoneal macrophages for cell culture differentiation studies were obtained by injecting intraperitoneally 1 ml of 3% Thioglycollate medium (Difco). Four days later, mice were sacrificed and peritoneal macrophages were collected from the abdominal cavity by washing with 5 ml PBS. Cells (1.4 × 10^6^ cells/ml) were dispensed onto 6-well plates (Corning Costar) and incubated at 37°C in 5% CO^2^ for 75 minutes. Non-adherent cells were discarded and RPMI-1640 (Gibco) containing 10% fetal calf serum (Biological Industries) was added. For M1 differentiation, 10 ng/ml GM-CSF and 10 ng/ml LPS (PeprotTech) were added to the culture medium followed by incubation at 37°C in 5% CO2 for 20 h. Alternatively, for M2 differentiation, 10 ng/ml M-CSF and 10 ng/ml IL-4 (PeprotTech) were added to the medium. [56] 200 nM PIF were added to the medium together with the differentiation factors.

### Real-time PCR analysis

Total colonic RNA was extracted using RNeasy^®^ Mini Kit columns (QIAGEN) according to the manufacturer's protocol. 1 μg of total RNA was used to synthesize cDNA using High-Capacity cDNA kits (Applied Biosystems) according to manufacturer's instructions. Detection of transcript levels of B7H1 and NOS2, was performed using the TaqMan Gene Expression Assay Kit (Applied Biosystems). HPRT-1 was used as a housekeeping gene transcript to normalize endogenous control. All primers were purchased from Applied Biosystems. Real-Time PCR reaction was carried out using the ABI Prism 7900 Sequence system (Applied Biosystems). Data were analyzed by StepOne Software version 2.2 (Applied Biosystems). The analysis of gene expression was calculated via a non-parametric Mann-Whitney *U* Test. *p* < 0.05 was considered significant.

### GI gene array

30 mg of jejunum tissue was excised and homogenized in a Fastprep 120 tissue homogenizer (30 s at 4.0 m/sec) in cell lysis buffer (Qiagen). Total RNA was extracted from cells using PureLink RNA Mini Kits (Ambion). Total RNA (250 ng) was amplified into cRNA using TotalPrep RNA amplification kits (Ambion) following manufacture's instruction. After amplification, 1.5 μg of cRNA was mixed with the hybridization controls and it was hybridized to MouseRef-8 array (Illumina). The array was hybridized for 16 h in a hybridization oven with a rocking platform at 58°C. The array chip then went through a series of washes before it was stained with streptavidin-Cy3. After the staining, it went through a final wash and drying. The array was scanned using the Illumina HiScan Scanner.

### Analysis of illumina gene array data

The raw hybridization data were processed using R with the Bioconductor lumi package. Data were first variance stabilized and then normalized using robust spline normalization. Both of these operations are used as implemented in the lumi package. Differential gene expression between all pairs of sample groups – i.e. radiation vs control, radiation with PIF treatment vs. control and radiation vs radiation with PIF treatment – was calculated using the moderated *t*-test as implemented in the Bioconductor limma package.

### Pathway analysis

The output of the limma analysis was used to perform gene set enrichment analysis (GSEA) using the SetRank method. The key principle of this algorithm is that it discards gene sets that have initially been flagged as significant, if their significance is only due to the overlap with another gene set. It calculates the *p*-value of a gene set using the ranking of its genes in the ordered list of *p*-values as calculated by limma. The following databases were searched for significant gene sets: BIOCYC. [57], Gene Ontology [58], ITFP [59], KEGG [60], LIPID MAPS [52], PhosphoSitePlus [53], REACTOME [61], and WikiPathways [62].

For the Heatmap the color scale indicates the relative rank of pathway in a given comparison, with 1.0 meaning top rank and 0.0 indicating a pathway that was not found significant at all. For the gene set network analysis every node represents a gene set. The size of a node reflects the number of genes in a gene set; the node color reflects the significance (corrected *p*-value) with lower *p*-values having a red color. Edges denote overlap (i.e. one or more shared genes) between two gene sets. The width of an edge reflects the relative size of the intersection (Jaccard similarity) compared to both gene sets. Edges pointed one way denote relationships where subtracting one gene set from a second reduces the significance of the latter. In this case, the arrow points towards the more significant gene set. Bidirectional edges indicate subset relations; double lines indicate cases where the significance of both gene sets is solely due to the intersection between these.

### Statistical analysis

Data from the 2 h post- 8 Gy lethal studies are described as mean values where the detailed individual values (WBC subtypes, RBC details and platelet count) are provided in the supplement section at every time point. Data from the remainder of the *in vivo* studies are represented as mean ± SEM. Data from *in vitro* studies are represented as mean ± SD. Single comparisons to control were made using two-tailed Student's *t*-test or Mann-Whitney test. One-way repeated measures ANOVA followed by Bonferroni's Multiple Comparison was used for data analysis, *p* < 0.05 was considered to be statistically significant. Ileum global gene analysis was carried out using heatmap followed by individual genes determining differences among the groups setting *p* < 0.05 as significant.

## SUPPLEMENTARY MATERIALS FIGURES AND TABLES



## References

[1] Chao NJ (2007). Accidental or intentional exposure to ionizing radiation: biodosimetry and treatment options. Exp Hematol.

[2] Donnelly EH, Nemhauser JB, Smith JM, Kazzi ZN, Farfan EB, Chang AS, Naeem SF (2010). Acute radiation syndrome: assessment and management. South Med J.

[3] Gilbert ES (2009). Ionising radiation and cancer risks: what have we learned from epidemiology?. Int J Radiat Biol.

[4] Dorr H, Meineke V (2011). Acute radiation syndrome caused by accidental radiation exposure - therapeutic principles. BMC Med.

[5] Herodin F, Mayol JF, Mourcin F, Drouet M (2005). Which place for stem cell therapy in the treatment of acute radiation syndrome?. Folia Histochem Cytobiol.

[6] Shan YX, Jin SZ, Liu XD, Liu Y, Liu SZ (2007). Ionizing radiation stimulates secretion of pro-inflammatory cytokines: dose-response relationship, mechanisms and implications. Radiat Environ Biophys.

[7] Waselenko JK, MacVittie TJ, Blakely WF, Pesik N, Wiley AL, Dickerson WE, Tsu H, Confer DL, Coleman CN, Seed T, Lowry P, Armitage JO, Dainiak N, Strategic National Stockpile Radiation Working G (2004). Medical management of the acute radiation syndrome: recommendations of the Strategic National Stockpile Radiation Working Group. Ann Intern Med.

[8] Bertho JM, Frick J, Prat M, Demarquay C, Dudoignon N, Trompier F, Gorin NC, Thierry D, Gourmelon P (2005). Comparison of autologous cell therapy and granulocyte-colony stimulating factor (G-CSF) injection vs. G-CSF injection alone for the treatment of acute radiation syndrome in a non-human primate model. Int J Radiat Oncol Biol Phys.

[9] Koenig KL, Goans RE, Hatchett RJ, Mettler FA, Schumacher TA, Noji EK, Jarrett DG (2005). Medical treatment of radiological casualties: current concepts. Ann Emerg Med.

[10] Azar Y, Shainer R, Almogi-Hazan O, Bringer R, Compton SR, Paidas MJ, Barnea ER, Or R (2013). Preimplantation factor reduces graft-versus-host disease by regulating immune response and lowering oxidative stress (murine model). Biol Blood Marrow Transplant.

[11] Kawakatsu M, Urata Y, Goto S, Ono Y, Li TS (2013). Placental extract protects bone marrow-derived stem/progenitor cells against radiation injury through anti-inflammatory activity. J Radiat Res.

[12] Lacave-Lapalun JV, Benderitter M, Linard C (2014). Flagellin and LPS each restores rat lymphocyte populations after colorectal irradiation. J Leukoc Biol.

[13] Mihaescu A, Thornberg C, Santen S, Mattsson S, Jeppsson B, Thorlacius H (2012). Radiation-induced platelet-endothelial cell interactions are mediated by P-selectin and P-selectin glycoprotein ligand-1 in the colonic microcirculation. Surgery.

[14] Semont A, Demarquay C, Bessout R, Durand C, Benderitter M, Mathieu N (2013). Mesenchymal stem cell therapy stimulates endogenous host progenitor cells to improve colonic epithelial regeneration. PLoS One.

[15] Bessout R, Demarquay C, Moussa L, Rene A, Doix B, Benderitter M, Semont A, Mathieu N (2015). TH17 predominant T cell responses in radiation-induced bowel disease are modulated by treatment with adipose-derived mesenchymal stromal cells. J Pathol.

[16] Kantara C, Moya SM, Houchen CW, Umar S, Ullrich RL, Singh P, Carney DH (2015). Novel regenerative peptide TP508 mitigates radiation-induced gastrointestinal damage by activating stem cells and preserving crypt integrity. Lab Invest.

[17] Barnea ER (2007). Applying Embryo-Derived Immune Tolerance to the Treatment of Immune Disorders. Annals of the New York Academy of Sciences.

[18] Barnea ER, Kirk D, Paidas MJ (2012). Preimplantation factor (PIF) promoting role in embryo implantation: increases endometrial integrin-alpha2beta3, amphiregulin and epiregulin while reducing betacellulin expression via MAPK in decidua. Reprod Biol Endocrinol.

[19] Duzyj CM, Barnea ER, Li M, Huang SJ, Krikun G, Paidas MJ (2010). Preimplantation factor promotes first trimester trophoblast invasion. Am J Obstet Gynecol.

[20] Duzyj CM, Paidas MJ, Jebailey L, Huang JS, Barnea ER (2014). PreImplantation Factor (PIF*) promotes embryotrophic and neuroprotective decidual genes: effect negated by epidermal growth factor. Journal of Neurodevelopmental Disorders.

[21] Moindjie H, Santos ED, Loeuillet L, Gronier H, de Mazancourt P, Barnea ER, Vialard F, Dieudonne MN (2014). Preimplantation factor (PIF) promotes human trophoblast invasion. Biol Reprod.

[22] Paidas MJ, Krikun G, Huang SJ, Jones R, Romano M, Annunziato J, Barnea ER (2010). A genomic and proteomic investigation of the impact of preimplantation factor on human decidual cells. Am J Obstet Gynecol.

[23] Stamatkin CW, Roussev RG, Stout M, Absalon-Medina V, Ramu S, Goodman C, Coulam CB, Gilbert RO, Godke RA, Barnea ER (2011). PreImplantation Factor (PIF) correlates with early mammalian embryo development-bovine and murine models. Reprod Biol Endocrinol.

[24] Barnea ER (2004). Insight into early pregnancy events: the emerging role of the embryo. Am J Reprod Immunol.

[25] Stamatkin CW, Roussev RG, Stout M, Coulam CB, Triche E, Godke RA, Barnea ER (2011). Preimplantation factor negates embryo toxicity and promotes embryo development in culture. Reprod Biomed Online.

[26] Barnea ER, Kirk D, Ramu S, Rivnay B, Roussev R, Paidas MJ (2012). PreImplantation Factor (PIF) orchestrates systemic antiinflammatory response by immune cells: effect on peripheral blood mononuclear cells. Am J Obstet Gynecol.

[27] Barnea ER, Kirk D, Todorova K, McElhinney J, Hayrabedyan S, Fernandez N (2015). PIF direct immune regulation: Blocks mitogen-activated PBMCs proliferation, promotes TH2/TH1 bias, independent of Ca(2+). Immunobiology.

[28] Roussev RG, Dons'koi BV, Stamatkin C, Ramu S, Chernyshov VP, Coulam CB, Barnea ER (2013). Preimplantation factor inhibits circulating natural killer cell cytotoxicity and reduces CD69 expression: implications for recurrent pregnancy loss therapy. Reprod Biomed Online.

[29] Mueller M, Schoeberlein A, Zhou J, Joerger-Messerli M, Oppliger B, Reinhart U, Bordey A, Surbek D, Barnea ER, Huang Y, Paidas M (2015). PreImplantation Factor bolsters neuroprotection via modulating Protein Kinase A and Protein Kinase C signaling. Cell Death Differ.

[30] Mueller M, Zhou J, Yang L, Gao Y, Wu F, Schoeberlein A, Surbek D, Barnea ER, Paidas M, Huang Y (2014). PreImplantation factor promotes neuroprotection by targeting microRNA let-7. Proc Natl Acad Sci USA.

[31] Weiss L, Bernstein S, Jones R, Amunugama R, Krizman D, Jebailey L, Almogi-Hazan O, Yekhtin Z, Shiner R, Reibstein I, Triche E, Slavin S, Or R (2011). Preimplantation factor (PIF) analog prevents type I diabetes mellitus (TIDM) development by preserving pancreatic function in NOD mice. Endocrine.

[32] Weiss L, Or R, Jones RC, Amunugama R, JeBailey L, Ramu S, Bernstein SA, Yekhtin Z, Almogi-Hazan O, Shainer R, Reibstein I, Vortmeyer AO, Paidas MJ (2012). Preimplantation factor (PIF*) reverses neuroinflammation while promoting neural repair in EAE model. J Neurol Sci.

[33] Chen YC, Rivera J, Fitzgerald M, Hausding C, Ying YL, Wang X, Todorova K, Hayrabedyan S, Barnea ER, Peter K (2016). PreImplantation factor prevents atherosclerosis via its immunomodulatory effects without affecting serum lipids. Thromb Haemost.

[34] Barnea ER, Almogi-Hazan O, Or R, Mueller M, Ria F, Weiss L, Paidas MJ (2015). Immune regulatory and neuroprotective properties of preimplantation factor: From newborn to adult. Pharmacol Ther.

[35] Barnea ER, Hayrabedyan S, Todorova K, Almogi-Hazan O, Or R, Guingab J, McElhinney J, Fernandez N, Barder TJ (2016). PIF Regulates Systemic Immunity and Targets Protective Regulatory and Cytoskeleton Proteins. Immunobiology.

[36] Barnea ER, Lubman DM, Liu YH, Absalon-Medina V, Hayrabedyan S, Todorova K, Gilbert RO, Guingab J, Barder TJ (2014). Insight into PreImplantation Factor (PIF*) mechanism for embryo protection and development: target oxidative stress and protein misfolding (PDI and HSP) through essential RIPK binding site. PLoS One.

[37] Chen YC, Rivera J, Fitzgerald M, Hausding C, Ying YL, Wang X, Todorova K, Hayrabedyan S, Barnea ER, Peter K (2016). PreImplantation factor prevents atherosclerosis via its immunomodulatory effects without affecting serum lipids. Thromb Haemost.

[38] Nunamaker EA, Artwohl JE, Anderson RJ, Fortman JD (2013). Endpoint refinement for total body irradiation of C57BL/6 mice. Comp Med.

[39] Shainer R, Azar Y, Almogi-Hazan O, Bringer R, Compton SR, Paidas MJ, Barnea ER, Or R (2013). Immune Regulation and Oxidative Stress Reduction by Preimplantation Factor following Syngeneic or Allogeneic Bone Marrow Transplantation. Conference Papers in Medicine.

[40] Barnea ER, Hayrabedyan S, Todorova K, Almogi-Hazan O, Or R, Guingab J, McElhinney J, Fernandez N, Barder T (2016). PreImplantation factor (PIF*) regulates systemic immunity and targets protective regulatory and cytoskeleton proteins. Immunobiology.

[41] Rube CE, Wilfert F, Uthe D, Konig J, Liu L, Schuck A, Willich N, Remberger K, Rube C (2004). Increased expression of pro-inflammatory cytokines as a cause of lung toxicity after combined treatment with gemcitabine and thoracic irradiation. Radiother Oncol.

[42] Fleetwood AJ, Dinh H, Cook AD, Hertzog PJ, Hamilton JA (2009). GM-CSF- and M-CSF-dependent macrophage phenotypes display differential dependence on type I interferon signaling. J Leukoc Biol.

[43] Williams CS, Mann M, DuBois RN (1999). The role of cyclooxygenases in inflammation, cancer, and development. Oncogene.

[44] Hasler R, Kerick M, Mah N, Hultschig C, Richter G, Bretz F, Sina C, Lehrach H, Nietfeld W, Schreiber S, Rosenstiel P (2011). Alterations of pre-mRNA splicing in human inflammatory bowel disease. Eur J Cell Biol.

[45] Uzhachenko R, Shanker A, Yarbrough WG, Ivanova AV (2015). Mitochondria, calcium, and tumor suppressor Fus1: At the crossroad of cancer, inflammation, and autoimmunity. Oncotarget.

[46] Black DL (2003). Mechanisms of alternative pre-messenger RNA splicing. Annu Rev Biochem.

[47] Mueller M, Schoeberlein A, Zhou J, Joerger-Messerli M, Oppliger B, Reinhart U, Bordey A, Surbek D, Barnea ER, Huang Y, Paidas M (2015). PreImplantation Factor bolsters neuroprotection via modulating Protein Kinase A and Protein Kinase C signaling. Cell Death Differ.

[48] Wang A, Keita AV, Phan V, McKay CM, Schoultz I, Lee J, Murphy MP, Fernando M, Ronaghan N, Balce D, Yates R, Dicay M, Beck PL (2014). Targeting mitochondria-derived reactive oxygen species to reduce epithelial barrier dysfunction and colitis. Am J Pathol.

[49] Douablin A, Deguillien M, Breig O, Baklouti F (2015). HnRNP A1 tethers KSRP to an exon splicing silencer that inhibits an erythroid-specific splicing event in PU. 1-induced erythroleukemia. Am J Cancer Res.

[50] Huai J, Vogtle FN, Jockel L, Li Y, Kiefer T, Ricci JE, Borner C (2013). TNFalpha-induced lysosomal membrane permeability is downstream of MOMP and triggered by caspase-mediated NDUFS1 cleavage and ROS formation. J Cell Sci.

[51] Tello D, Balsa E, Acosta-Iborra B, Fuertes-Yebra E, Elorza A, Ordonez A, Corral-Escariz M, Soro I, Lopez-Bernardo E, Perales-Clemente E, Martinez-Ruiz A, Enriquez JA, Aragones J (2011). Induction of the mitochondrial NDUFA4L2 protein by HIF-1alpha decreases oxygen consumption by inhibiting Complex I activity. Cell Metab.

[52] Fahy E, Subramaniam S, Murphy RC, Nishijima M, Raetz CR, Shimizu T, Spener F, van Meer G, Wakelam MJ, Dennis EA (2009). Update of the LIPID MAPS comprehensive classification system for lipids. J Lipid Res.

[53] Hornbeck PV, Kornhauser JM, Tkachev S, Zhang B, Skrzypek E, Murray B, Latham V, Sullivan M (2012). PhosphoSitePlus: a comprehensive resource for investigating the structure and function of experimentally determined post-translational modifications in man and mouse. Nucleic Acids Res.

[54] Chargari C, Riet F, Mazevet M, Morel E, Lepechoux C, Deutsch E (2013). Complications of thoracic radiotherapy. Presse Med.

[55] Hinden L, Shainer R, Almogi-Hazan O, Or R (2015). *Ex Vivo* Induced Regulatory Human/Murine Mesenchymal Stem Cells as Immune Modulators. Stem Cells.

[56] Qin H, Holdbrooks AT, Liu Y, Reynolds SL, Yanagisawa LL, Benveniste EN (2012). SOCS3 deficiency promotes M1 macrophage polarization and inflammation. J Immunol.

[57] Karp PD, Ouzounis CA, Moore-Kochlacs C, Goldovsky L, Kaipa P, Ahren D, Tsoka S, Darzentas N, Kunin V, Lopez-Bigas N (2005). Expansion of the BioCyc collection of pathway/genome databases to 160 genomes. Nucleic Acids Res.

[58] Ashburner M, Ball CA, Blake JA, Botstein D, Butler H, Cherry JM, Davis AP, Dolinski K, Dwight SS, Eppig JT, Harris MA, Hill DP, Issel-Tarver L (2000). Gene ontology: tool for the unification of biology. The Gene Ontology Consortium. Nat Genet.

[59] Zheng G, Tu K, Yang Q, Xiong Y, Wei C, Xie L, Zhu Y, Li Y (2008). ITFP: an integrated platform of mammalian transcription factors. Bioinformatics.

[60] Kanehisa M, Goto S, Sato Y, Kawashima M, Furumichi M, Tanabe M (2014). Data, information, knowledge and principle: back to metabolism in KEGG. Nucleic Acids Res.

[61] Croft D, Mundo AF, Haw R, Milacic M, Weiser J, Wu G, Caudy M, Garapati P, Gillespie M, Kamdar MR, Jassal B, Jupe S, Matthews L (2014). The Reactome pathway knowledgebase. Nucleic Acids Res.

[62] Kelder T, van Iersel MP, Hanspers K, Kutmon M, Conklin BR, Evelo CT, Pico AR (2012). WikiPathways: building research communities on biological pathways. Nucleic Acids Res.

