# Supplementary material for: PreImplantation factor (PIF) therapy provides comprehensive protection against radiation induced pathologies
Source: Oncotarget. 2016 Jul 16;7(37):58975–94. doi: 10.18632/oncotarget.10635 (PMC5312289; doi:10.18632/oncotarget.10635)
Supplement: Supplementary file 1 [file oncotarget-07-58975-s001.pdf]

PreImplantation factor (PIF) therapy provides comprehensive protection against radiation induced pathologies

Supplementary Materials

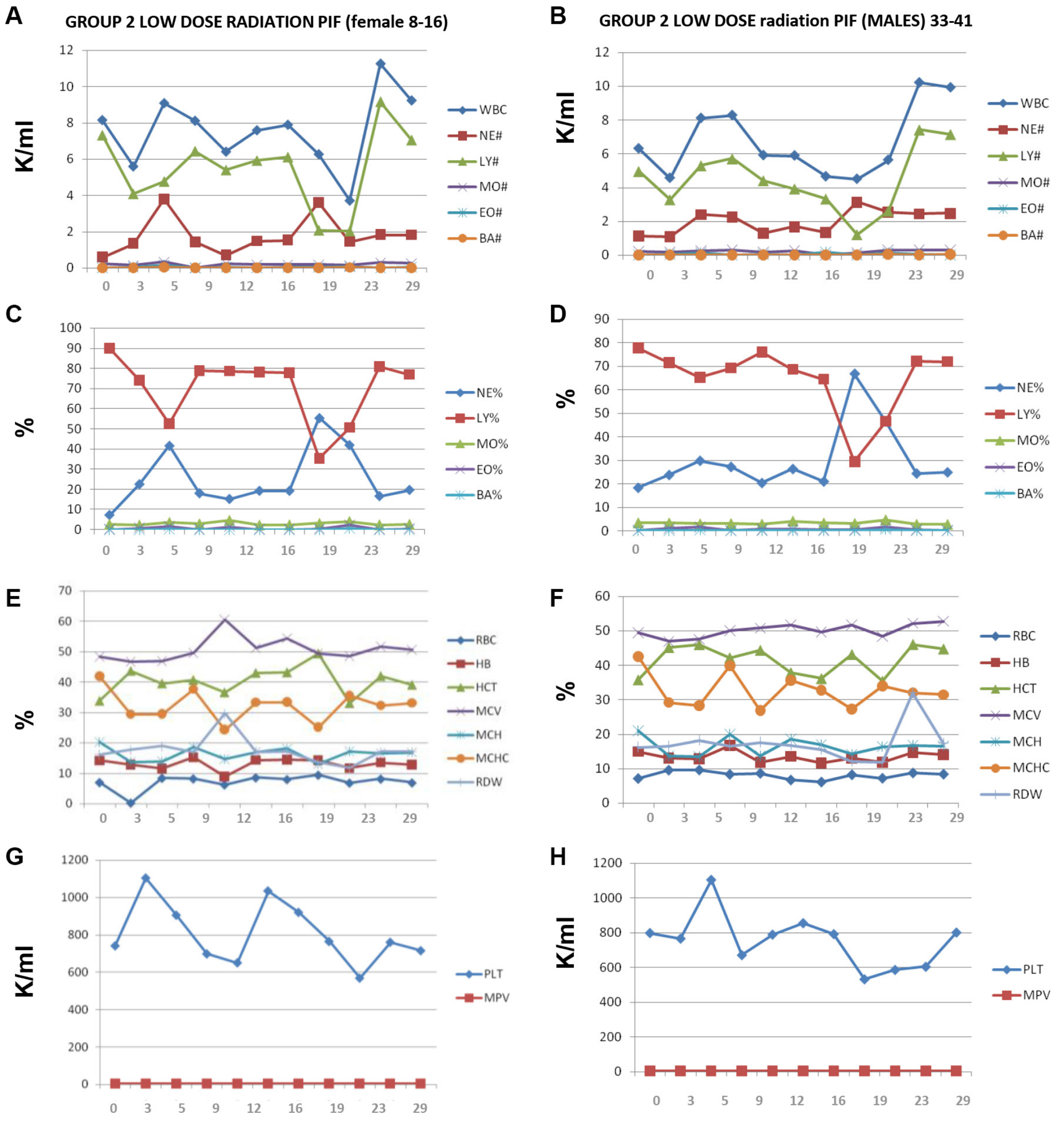

Supplementary Figure S1: PIF protective effect on blood indices of lethally radiated (800 rads) mice. 2 h post radiation PIF was administered BID up to 14 days followed by 14 days follow up. A,C,E,F are WBC, red blood and platelets indices of low dose PIF treated females. B,D,F,H PIF low dose treated males.

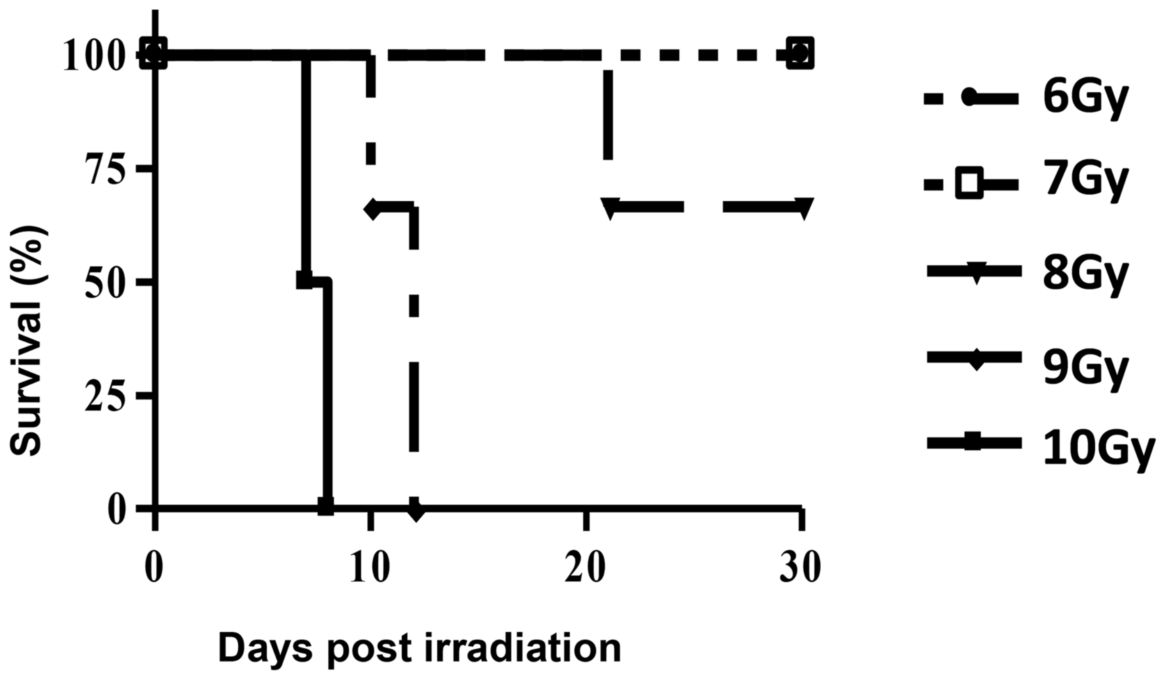

Supplementary Figure S2: mice 10/group were exposed to increasing doses of radiation to determine LD. All mice died by day 11 after exposure to 10 or 9 Gy. By 8 Gy LD 70/30 was observed and by 7–6 Gy exposure all mice survived.

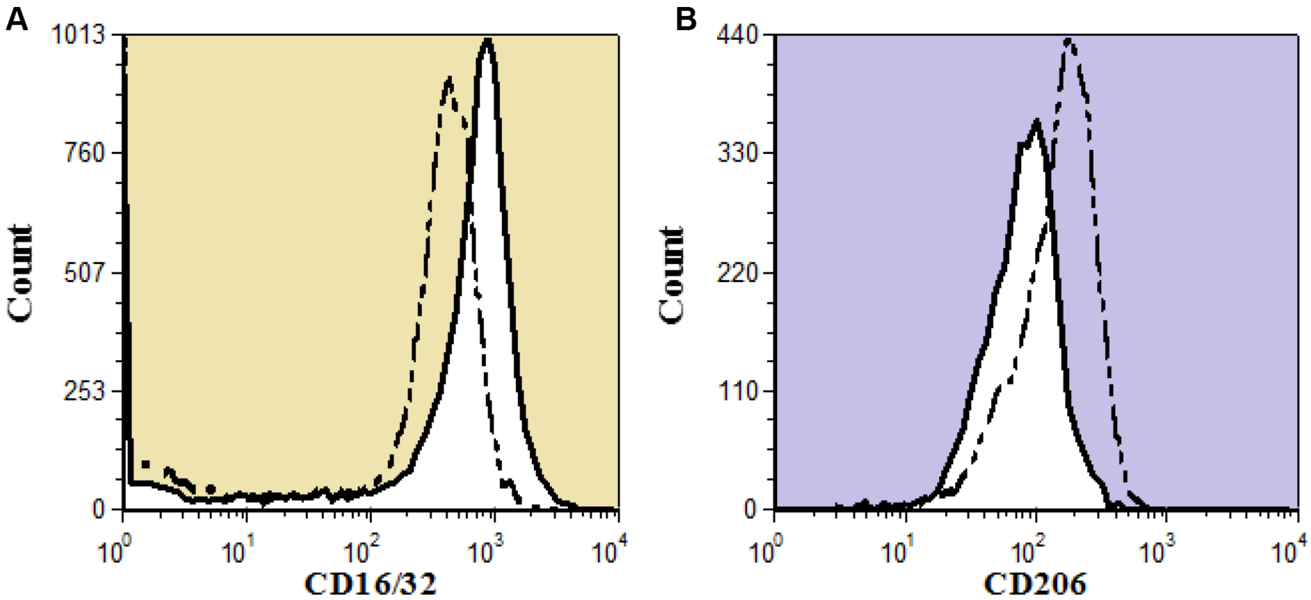

Supplementary Figure S3: M1/M2 phenotvpes. Peritoneal macrophages were cultured with GM- CSF (10 ng/ml) and LPS (10 ng/ml) for Ml differentiation or with M-CSF (10 ng/ml) and IL-4 (10 ng/ml) for M2 differentiation for 2øh. FACS analysis of CD 16/32 (**A**) and CD206 (**B**). Solid line represent Ml and tlie dashed line represent M2 macrophages. One representative figure of four independent experiments.

Supplementary Table S1: PIF effect on the global GI genome was determined and compared with PBS (vehicle) treated mice after 24 hours exposed to sublethal- 600 rads

PIF induced upregulation of top 20 GI genes expression after 6 Gy exposure

GENE	DESCRIPTION	MW	FOLD CHANGE	*P* VALUE	ADJ *P* VALUE
Cfd	Mus musculus complement factor D (adipsin) (Cfd), mRNA.	11537	0.858	1.57E–001	9.67E–001
Olfm4	Mus musculus olfactomedin 4 (Olfm4), mRNA.	380924	0.799	1.00E–001	9.67E–001
Lyz1	Mus musculus lysozyme 1 (Lyz1), mRNA.	17110	0.733	3.20E–001	9.67E–001
Scd1	Mus musculus stearoyl–Coenzyme A desaturase 1 (Scd1), mRNA.	20249	0.730	1.21E–001	9.67E–001
Rnase1	Mus musculus ribonuclease, RNase A family, 1 (pancreatic) (Rnase1), mRNA.	19752	0.715	8.96E–002	9.67E–001
Adh1	Mus musculus alcohol dehydrogenase 1 (class I) (Adh1), mRNA.	11522	0.714	3.37E–001	9.67E–001
Reg1	Mus musculus regenerating islet-derived 1 (Reg1), mRNA.	19692	0.679	6.32E–001	9.75E–001
Cyp3a11	Mus musculus cytochrome P450, family 3, subfamily a, polypeptide 11 (Cyp3a11), mRNA.	13112	0.654	2.30E–001	9.67E–001
Casp6	Mus musculus caspase 6 (Casp6), mRNA.	12368	0.647	1.20E–001	9.67E–001
Ccl5	Mus musculus chemokine (C-C motif) ligand 5 (Ccl5), mRNA.	20304	0.647	8.73E–004	9.67E–001
Acaa2	Mus musculus acetyl-Coenzyme A acyltransferase 2 (mitochondrial 3-oxoacyl-Coenzyme A thiolase) (Acaa2), nuclear gene encoding mitochondrial protein, mRNA.	52538	0.618	1.39E–001	9.67E–001
Rps3	Mus musculus ribosomal protein S3 (Rps3), mRNA.	27050	0.610	2.19E–002	9.67E–001
Khk	Mus musculus ketohexokinase (Khk), mRNA.	16548	0.600	4.04E–002	9.67E–001
Mbl2	Mus musculus mannose-binding lectin (protein C) 2 (Mbl2), mRNA.	17195	0.595	2.21E–002	9.67E–001
Atp5f1	Mus musculus ATP synthase, H+ transporting, mitochondrial F0 complex, subunit b, isoform 1 (Atp5f1), mRNA.	11950	0.586	1.52E–001	9.67E–001
Scye1	Mus musculus small inducible cytokine subfamily E, member 1 (Scye1), mRNA.	13722	0.574	1.82E–002	9.67E–001
Sep15	Mus musculus selenoprotein (Sep15), mRNA.	93684	0.551	1.79E–001	9.67E–001
Ppp1ca	Mus musculus protein phosphatase 1, catalytic subunit, alpha isoform (Ppp1ca), mRNA.	19045	0.549	2.28E–001	9.67E–001
Cox7a2l	Mus musculus cytochrome c oxidase subunit VIIa polypeptide 2-like (Cox7a2l), mRNA.	20463	0.539	5.23E–002	9.67E–001
Clca6	Mus musculus chloride channel calcium activated 6 (Clca6), mRNA.	99663	0.527	1.26E–002	9.67E–001

Following sacrifice the tissue was extracted mRNA generated and ran on an Illumina chip. (N = 7/group). Data generated was analyzed determining top ranking genes. Further details are described in the method section.

Supplementary Table S2: PIF effect on the global GI genome was determined and compared with PBS (vehicle) treated mice after 24 hours exposed to sublethal- 600 rads

GENE	DESCRIPTION	MW	FOLD CHANGE	*P* VALUE	ADJ *P* VALUE
Fabp6	Mus musculus fatty acid binding protein 6, ileal (gastrotropin) (Fabp6), mRNA.	16204	–3.060	3.83E–002	9.67E–001
LOC100046120	PREDICTED: Mus musculus similar to clusterin (LOC100046120), mRNA.	NA	–1.054	3.58E–002	9.67E–001
Tmem117	Mus musculus transmembrane protein 117 (Tmem117), mRNA.	320709	–0.788	2.03E–001	9.67E–001
Serpina1b	Mus musculus serine (or cysteine) peptidase inhibitor, clade A, member 1b (Serpina1b), mRNA.	20701	–0.721	1.35E–001	9.67E–001
Prss7	Mus musculus protease, serine, 7 (enterokinase) (Prss7), transcript variant 1, mRNA.	19146	–0.681	2.68E–001	9.67E–001
Fam151a	Mus musculus family with sequence simliarity 151, member A (Fam151a), mRNA.	230579	–0.627	1.58E–001	9.67E–001
Pcsk9	Mus musculus proprotein convertase subtilisin/kexin type 9 (Pcsk9), mRNA.	100102	–0.610	5.22E–002	9.67E–001
Cfl2	Mus musculus cofilin 2, muscle (Cfl2), mRNA.	12632	–0.587	3.51E–001	9.67E–001
Mfge8	Mus musculus milk fat globule-EGF factor 8 protein (Mfge8), transcript variant 2, mRNA	17304	–0.579	4.13E–002	9.67E–001
Ccl21a	Mus musculus chemokine (C-C motif) ligand 21A (Ccl21a), mRNA.	18829	–0.576	1.35E–001	9.67E–001
Slc5a6	Mus musculus solute carrier family 5 (sodium-dependent vitamin transporter), member 6 (Slc5a6), mRNA.	330064	–0.557	7.03E–002	9.67E–001
Xpnpep2	Mus musculus X-prolyl aminopeptidase (aminopeptidase P) 2, membrane-bound (Xpnpep2), transcript variant 1, mRNA.	170745	–0.555	1.17E–001	9.67E–001
Ddah1	Mus musculus Dimethylargininase-1 , mRNA	69219	–0.554	2.67E–001	9.67E–001
Cxcl13	Mus musculus chemokine (C-X-C) motif 13, mRNA	55985	–0.535	4.91E–002	9.67E–001
Aim1	Mus musculus suppressor of tumorigenicity 4, mRNA	11630	–0.480	1.18E–002	9.67E–001
Gm766	Mus musculus gene model 766, (NCBI) (Gm766), mRNA.	330440	–0.502	3.75E–001	9.67E–001
Serpina1b	Mus musculus serine (or cysteine) peptidase inhibitor, clade A, member 1b (Serpina1b), mRNA.	20701	–0.497	1.07E–001	9.67E–001
Osta	Mus musculus organic solute transporter alpha (Osta), mRNA.	106407	–0.491	5.77E–002	9.67E–001
Panx1	Mus musculus pannexin 1 (Panx1), mRNA.	55991	–0.490	4.65E–003	9.67E–001
Zmiz1	Mus musculus zinc finger, MIZ-type containing 1 (Zmiz1), mRNA.	328365	–0.488	2.21E–002	9.67E–001

Following sacrifice the tissue was extracted mRNA generated and ran on an Illumina chip. (N = 7/group). Data generated was analyzed determining most down-regulated genes by PIF. Further details are described in the method section.

Supplementary Table S3: PIF is currently being used in Phase Ib clinical trial

Assay and Impurities data of sPIF (15 mg/vial) at ambient conditions

Lyophilized vial Lot #: 0127–13106–3

Time point	Assay		Total Impurities	U–1	U–2	U–3	U–4	U–5
	% Label	% Area	% Area	0.97	0.99	1.04	1.08	1.13
Time Zero	100.7	98.4	1.6	0.22		1.15	0.26	
8 weeks	110.9	96.1	3.9	0.31		3.56		
	111.1	95.9	4.1	0.37	0.11	3.60		

Suitable for deployment in field use military or civilian. The stability of PIF was tested by keeping in ambient temperature for 2 m. No significant degradation was noted > 95% purity.
